# Evaluation of maternal and child care continuum in Guinea: a secondary analysis of two demographic and health surveys using the composite coverage index (CCI)

**DOI:** 10.1186/s12884-023-05718-y

**Published:** 2023-05-27

**Authors:** Diao Cisse, Almamy Amara Toure, Abdourahamane Diallo, Juste Aristite Goungounga, Kadio Jean-Jacques Olivier Kadio, Ibrahima Barry, Souleymane Berete, Aboubacar Sidiki Magassouba, Souleymane Hassane Harouna, Alseny Yarie Camara, Younoussa Sylla, Kola Cisse, Maïmouna Sidibe, Abdoulaye Toure, Alexandre Delamou

**Affiliations:** 1 Department of Public Health, Faculty of Health Sciences and Techniques, Gamal Abdel Nasser University, Conakry, Guinea; 2 Medécins Sans Frontières Belgique, Conakry, Guinea; 3 National Centre for Training and Research in Rural Health (CNFRSR) of Maferinyah, Forécariah, Guinea; 4 Centre Hospitalo-Universitaire Ignace Deen, Service de Gynécologie, Conakry, Guinée; 5 Univ Rennes, EHESP, CNRS, Inserm, Arènes-UMR 6051, RSMS-U 1309, F-35000, Rennes, France; 6 Écoles Des Hautes Études en Santé Publique, Département METIS, 15 Avenue du Professeur Léon Bernard, CS 74312, 35043, Rennes Cedex, France; 7 Centre de Recherche Et de Formation en Infectiologie de Guinée, Conakry, Guinea; 8 Catholic Relief Service, Conakry, Guinea; 9 Médecins Sans Frontière Espagne, Bamako, Mali; 10 Centre Hospitalo-Universitaire Fann, Service de Maladies Infectieuses et Tropicales, Dakar, Sénégal; 11 Centre d´Excellence Africain pour la Prévention et le Contrôle des Maladies Transmissibles (CEA-PCMT), Gamal Abdel Nasser University, Conakry, Guinea

**Keywords:** Composite coverage index, Maternal and child health, Care continuum, DHS, Guinea

## Abstract

**Introduction:**

The composite coverage index (CCI) is the weighted average coverage of eight preventive and curative interventions received along the maternal and childcare continuum. This study aimed to analyse maternal and child health indicators using CCI.

**Methods:**

We performed a secondary analysis of demographic and health surveys (DHS) focused on women aged 15 to 49 and their children aged 1 to 4. This study took place in Guinea. The CCI (meeting the need for planning, childbirth assisted by qualified healthcare workers, antenatal care assisted by qualified healthcare workers, vaccination against diphtheria, pertussis, tetanus, measles and Bacillus Calmette-Guérin, taking oral rehydration salts during diarrhoea and seeking care for pneumonia) is optimal if the weighted proportion of interventions is > 50%; otherwise, it is partial. We identified the factors associated with CCI using the descriptive association tests, the spatial autocorrelation statistic and multivariate logistic regression.

**Results:**

The analyses involved two DHS surveys, with 3034 included in 2012 and 4212 in 2018. The optimal coverage of the CCI has increased from 43% in 2012 to 61% in 2018. In multivariate analysis, in 2012: the poor had a lower probability of having an optimal CCI than the richest; OR = 0.11 [95% CI; 0.07, 0.18]. Those who had done four antenatal care visits (ANC) were 2.78 times more likely to have an optimal CCI than those with less OR = 2.78 [95% CI;2.24, 3.45]. In 2018: the poor had a lower probability of having an optimal CCI than the richest OR = 0.27 [95% CI; 0.19, 0.38]. Women who planned their pregnancies were 28% more likely to have an optimal CCI than those who had not planned OR = 1.28 [95% CI;1.05, 1.56]. Finally, women with more than 4 ANC were 2.43 times more likely to have an optimal CCI than those with the least OR = 2.43 [95% CI; 2.03, 2.90]. The spatial analysis reveals significant disparities with an aggregation of high partial CCI in Labé between 2012 and 2018.

**Conclusion:**

This study showed an increase in CCI between 2012 and 2018. Policies should improve access to care and information for poor women. Besides, strengthening ANC visits and reducing regional inequalities increases optimal CCI.

**Supplementary Information:**

The online version contains supplementary material available at 10.1186/s12884-023-05718-y.

## Introduction

According to the World Health Organization (WHO), 295,000 women died in 2017 because of complications from pregnancy and childbirth [1]. Among these deaths, Sub-Saharan Africa and South Asia accounted for 254,000 [1]. Likewise, according to the WHO report, more than 5 million children died before their fifth birthday in 2020 [2] and Sub-Saharan Africa has the highest mortality risk [2]. With this in mind, monitoring maternal and child health indicators to assess progress made within the Sustainable Development Goals (SDG) framework is essential. These SDGs focus on reducing maternal and child mortality to less than 70 per 100,000 and less than 25 per 1,000 live births [3]. The maternal and child health indicators are numerous and sometimes complex to interpret when isolated; the WHO also reports this variety in the fundamental health indicators [4]. A solution to overcome this deficiency is to aggregate them; the composite coverage index (CCI) fills this need, the weighted average coverage of eight preventive and curative interventions received along the maternal and childcare continuum [5, 6]. These are the following indicators: meeting the need for planning, childbirth assisted by qualified healthcare workers, antenatal care visits assisted by qualified personnel, vaccination against the following diseases: diphtheria, pertussis and tetanus (DTC3), measles and vaccination against Bacillus Calmette-Guérin (BCG), taking oral rehydration salts during diarrhoea (ORT) and seeking care for pneumonia [5]. The CCI gives equal weight to family planning, maternal and newborn care, vaccination and the management of sick children and is an effective way to summarise and compare the coverage of maternal, newborn and child health interventions (MNCH) between countries and over time [7–9].

In Guinea, the Demographic and health survey (DHS) indicates progress in evolving specific maternal and child health indicators [10, 11]. Between 2012 and 2018, the proportion of satisfied family planning requests was 19.1% and 33%, respectively; 45.3% and 55.3% of women were attended by skilled health workers in 2012 and 2018, respectively [10, 11]. Regarding children's health, 37% and 83% benefited from seeking care during cough in 2012 and 2018, respectively; and 36.5% and 64% benefited from oral rehydration therapy during diarrhoea in 2012 and 2018 [10, 11]. Besides, other indicators experienced an apparent regression, so for BCG, DTC3 and measles, we noted 82.4%, 49.8% and 61.8% in 2012 against 71.4%, 40.9% and 46.1% in 2018 [10, 11]. Because of these different figures, it seems evident that a comparative and standardised analysis cannot be easy to implement, hence the interest in using the CCI. This index is synthetic, easy to use and optimises data analysis and interpretation efforts [8, 12]. Although the CCI has undergone some revisions recently [13], the initial index is still of apparent interest.

It is essential to use simple and practical approaches to monitor maternal and child health better and guide improvement efforts in the context of scarce resources. The analysis of maternal and child health indicators in Guinea using the CCI aims to inform policies on the progress achieved and the efforts to accelerate the achievement of the SDGs for mothers and their children.

## Methodology

### Study setting

Guinea is a West African country located in the Northern latitudes of 7°30' and 12°30', Western longitudes: of 8° and 15°; it is limited by Guinea-Bissau, Senegal, Mali, Ivory Coast, Sierra Leone, and Liberia [14]. Guinea homed 13,261,638 inhabitants in 2022. It has a gross birth rate of 33.6 per 1000, an infant mortality rate of 66 per 1000 and a life expectancy of 59 years [14].

### Design, study population and selection criteria

We analysed the secondary data from Guinea's fourth and fifth Demographic and Health Survey (DHS 2012 and 2018). These surveys covered the entire national territory. We included women aged 15 to 49 who gave birth in the last five years before the surveys and their children aged 1 to 4. We excluded the deceased children because of the lack of information on the age variables and vaccines.

### Summary of the sampling technique used by the DHS

The 2012 and 2018 DHS samples are stratified random samples drawn at two degrees [10, 11]. They drew Clusters throughout the national territory regarding the general population and housing census; for the 2012 and 2018 DHS, 300 and 401 clusters were selected at the first stage, and then clusters chose with a probability proportional to their size. In each cluster, a list of households was selected from which a sample in the second degree with systematic random.

### Variables

#### In this study, we were interested in the following variables

**Dependent variable:**

**•** CCI: Composed of eight established interventions, it includes the following indicators: the request for satisfied family planning (FPS), childbirth assisted by qualified healthcare workers (SBA), at least one antenatal care visit (ANCs) with a healthcare worker, three doses of DTC3 which is the equivalent of pentavalent in the Guinean context; these are five vaccines: diphtheria, tetanus, whooping cough, hepatitis B and Haemophilus influenza infections, measles vaccination (MSL), vaccination against bacillus Calmette – Guérin (BCG), oral rehydration therapy for children suffering from diarrhoea (ORT) and the search for care for pneumonia (CPNM) [15]. The formula below expresses the CCI:

$$CCI=\frac{1}{4}\left(FPS+\frac{SBA+ANCS}{2}+\frac{2DTC3+MSL+BCG}{4}+\frac{ORT+CPNM}{2}\right)$$

**Definitions of the component of the dependent variable:**

* **Childbirth assisted by qualified healthcare worker**: skilled healthcare worker (midwife, nurse-birth attendant, or doctor) who, thanks to the training he has received, can safely perform routine deliveries and detect complications very early to manage them or refer them.

* **Antenatal care by a qualified provider**: skilled healthcare workers (doctors, nurses or midwives) provide antenatal care.

* **Calmette-Guérin bacillus vaccination (1 dose)**: it is necessary to specify whether the child has received the tuberculosis vaccine.

* **Pentavalent:** it is necessary to specify whether the child had received the vaccine against diphtheria, tetanus, whooping cough, hepatitis B, and Haemophilus influenza infections (3 doses).

* **Measles vaccination (1 dose)**: to specify whether the child had received the measles vaccine.

* **Oral rehydration during diarrhoea.** To specify whether the child with diarrhoea had benefited from oral rehydration therapy.

* **Seeking care for pneumonia.** To specify whether the coughing child had benefited from the search for care for pneumonia.

* **Satisfied demand for family planning:** women of childbearing age (15 to 49 years old) whose need for family planning is satisfied with modern methods.

After weighing the dependent variable, we dichotomised it. We assumed partial coverage if CCI is < 0.50 and optimal coverage if CCI is ≥ 0.50.

#### Independent variables

##### Sociodemographic variables

The year of survey, age of the child, sex of the child, age of the mother, mother's education, marital status of the mother, education of the partner, whether the mother is currently working, type of residence, place of residence, administrative region, religion, ethnicity, age of the head of household, sex of the head of household, number of people in the household, access to the newspapers, access to the TV and access to the radio then economic variable (wealth quintile) and obstetric variables (number of antenatal care visits (ANCs), time of the first ANC and planned pregnancy).

### Statistical analysis

#### Univariate and bivariate analysis

The quantitative variables describe as median with the interquartile intervals, and the categorical variables as percentages. We checked the association between the dependent and independent variables using the Wilcoxon rank-sum test for complex sampling surveys and Rao & Scott's chi-squared second-order correction test [16].

#### Multivariate analysis

We used multivariate logistic regression to identify the factors associated with the optimal use of CCI. For selecting the relevant variables for the model, we used a simple top-down step-by-step procedure starting from the model containing all the independent variables and respecting the Akaike Information Criteria (AIC) minimisation criterion. The Hosmer–Lemeshow test was used to determine the quality of the fit of our regression model. Then, to select a profile of predictors, the variables of the final model of the multivariate logistic regression were used in the classification and regression tree (CART) while keeping the dependent variable on the multivariate logistic regression.

#### Spatial analysis

We portrayed the spatial distribution of the CCI according to the regions and the 2012 and 2018 DHS. We used the spatial autocorrelation statistic (Global Moran's I) to confirm or not the autocorrelation [17]. The information for the shapefiles has been downloaded from Spatial Data Repository-Boundaries (https://spatialdata.dhsprogram.com/boundaries/#view=table&countryId=AF).

We evaluated the existence of a local autocorrelation (LISA) using the local Moran index, which verifies the region's value with that of its neighbours. The observations have values higher than the variable's mean in a neighbourhood resembling them. It is a positive spatial autocorrelation with a high index value); low-low (the observations have values lower than the variable's mean in a neighbourhood that resembles them. It is a positive spatial autocorrelation with a low index value, high-low (observations have values higher than the variable's mean in a neighbourhood that does not resemble them [18]. It is a negative spatial autocorrelation with a high index value); low–high (observations have lower than average variable values in a neighbourhood that does not resemble them). It is a negative spatial autocorrelation with a low index value) [18].

#### Classification and regression tree (CART)

We use the Breiman method, classification and regression tree (CART) [19], using the R party package with the "*ctree"* function [20]. The final multivariate logistic regression model variables were used while keeping the variable dependent on the multivariate logistic regression.

All analyses were weighted to consider the complex sampling plan of the surveys [21]. We used the command "*svydesign*" in R [22]. The statistical tests were carried out at risk α = 5%. All p values < 0.05 were considered significant. Lastly, we did all the analyses using the R software version 4.1.3.

## Results

**Fig. 1:**
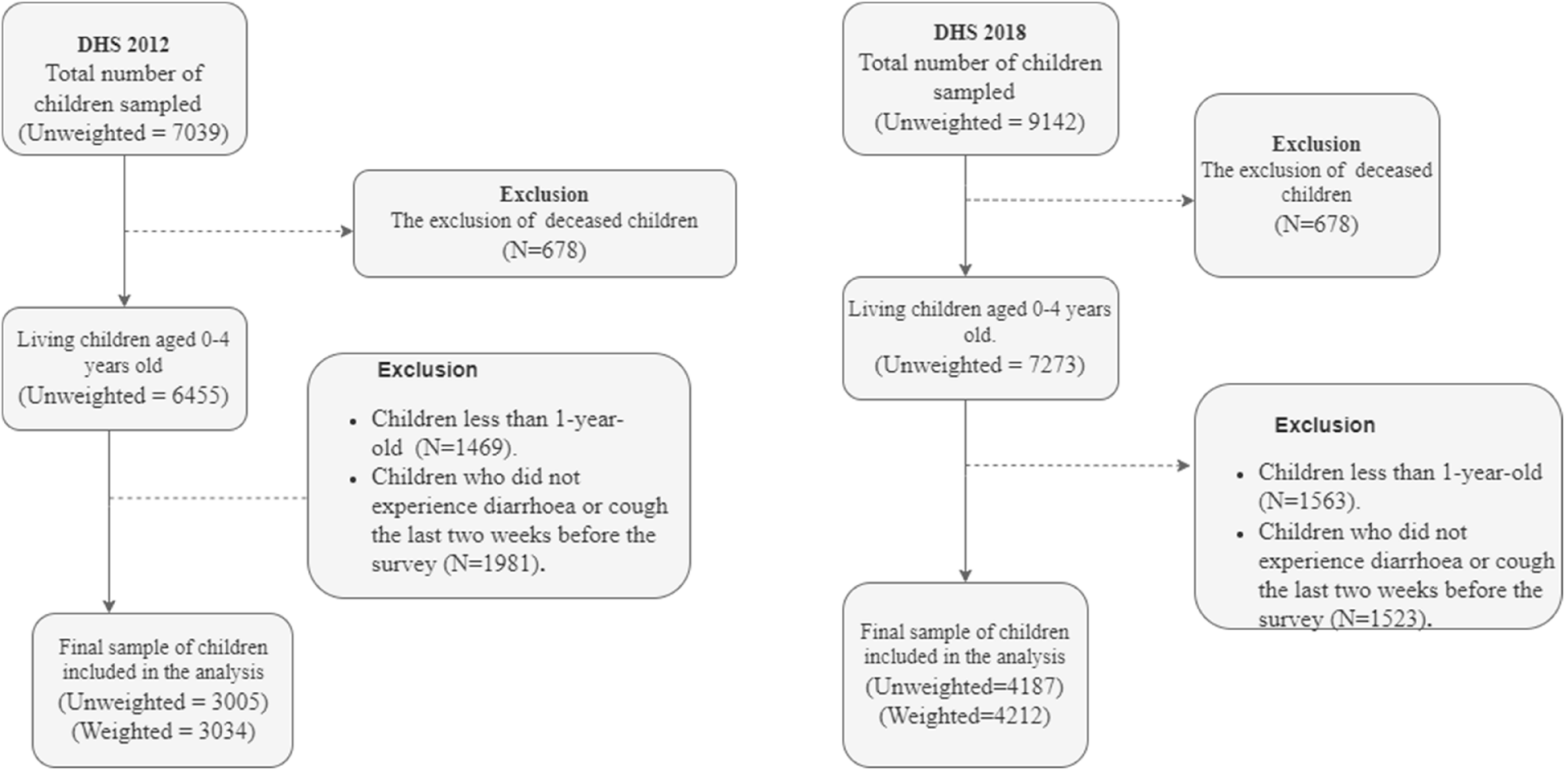
Inclusion flowchart for DHS 2012 and 2018

Figure 1 shows the inclusion diagram of the 2012 and 2018 DHS. A total of 3034 and 4212 mothers and their children were included in the study in 2012 and 2018, respectively.

### Univariate descriptive analysis

Table 1 reports the description of the study sample. For women who had at least one prenatal visit with a qualified provider, 74% benefited from it in 2012 compared to 84% in 2018. Similarly, 46% profited from childbirth assisted by qualified personnel in 2012 compared to 62% in 2018. Only 8.9% of family planning requests were satisfied compared to 16% in 2018. Regarding children, 56% received pentavalent in 2012 compared to 40% in 2018. Regarding the measles vaccine, 72% of children received it in 2012 compared to 45% in 2018. Similarly, 89% of children received BCG in 2012 compared to 67% in 2018. The percentage of children who received oral rehydration during diarrhoea increased from 60% in 2012 to 80% in 2018. Finally, 75% of children benefited from pneumonia seeking care in 2012 compared to 62% in 2018. Overall, the optimal coverage of CCI increased from 43% in 2012 to 61% in 2018 (Table 2).

**Table 1:** Characteristics of women aged 15-49 and their children under 5 in Guinea between DHS 2012 and 2018

Characteristics	DHS 2012 (*N* = 3034)	DHS 2018 (*N* = 4212)
Sociodemographic variables		
Age of mothers		
15–18	166 (5.5%)	185 (4.4%)
19–30	1,817 (60%)	2,408 (57%)
31–40	879 (29%)	1,335 (32%)
41–49	172 (5.7%)	285 (6.8%)
Administrative region		
Boké	308 (10%)	412 (9.8%)
Conakry	522 (17%)	592 (14%)
Faranah	309 (10%)	367 (8.7%)
Kankan	498 (16%)	827 (20%)
Kindia	472 (16%)	665 (16%)
Labé	225 (7.4%)	387 (9.2%)
Mamou	163 (5.4%)	309 (7.3%)
N’Zérékoré	538 (18%)	652 (15%)
Residence		
Rural	2,097 (69%)	2,788 (66%)
Urban	937 (31%)	1,425 (34%)
Religion		
No religion	132 (4.3%)	75 (1.8%)
Christian	277 (9.1%)	456 (11%)
Muslim	2,626 (87%)	3,681 (87%)
Ethnic		
Guerzé	166 (5.5%)	176 (9.7%)
Kissi	181 (6.0%)	102 (5.6%)
Malinké	1,008 (33%)	597 (33%)
Peulh	955 (31%)	508 (28%)
Soussou	563 (19%)	413 (23%)
Toma	79 (2.6%)	-
Others	82 (2.7%)	18 (1.0%)
Marital status		
Married	2,778 (92%)	3,947 (94%)
Single	256 (8.4%)	265 (6.3%)
Number of people in the household		
1–5	858 (28%)	1,226 (29%)
6–10	1,443 (48%)	2,194 (52%)
> 10	734 (24%)	792 (19%)
Education of the mother		
Higher	55 (1.8%)	98 (2.3%)
No education	2,252 (74%)	3,204 (76%)
Primary	403 (13%)	413 (9.8%)
Secondary	324 (11%)	497 (12%)
Sex of the head of the household		
Female	423 (14%)	517 (12%)
Male	2,611 (86%)	3,695 (88%)
Age of the head of household		
16–25	88 (2.9%)	130 (3.1%)
26–40	1,138 (37%)	1,684 (40%)
41–60	1,270 (42%)	1,837 (44%)
> 60	538 (18%)	562 (13%)
Access to newspapers		
Access	194 (6.4%)	291 (6.9%)
No access	2,840 (94%)	3,921 (93%)
Access to the radio		
Access	1,914 (63%)	2,652 (63%)
No access	1,120 (37%)	1,561 (37%)
Access to television		
Access	1,243 (41%)	1,892 (45%)
No access	1,791 (59%)	2,320 (55%)
Number of living children		
1–2	1,214 (40%)	1,558 (37%)
3–4	981 (32%)	1,556 (37%)
5–7	745 (25%)	968 (23%)
> 7	95 (3.1%)	130 (3.1%)
Education of the mothers’ partner		
Higher	225 (7.4%)	327 (7.8%)
No education	1,948 (64%)	2,957 (70%)
Primary	407 (13%)	307 (7.3%)
Secondary	454 (15%)	620 (15%)
Currently working		
Yes	2,431 (80%)	3,057 (73%)
No	603 (20%)	1155 (27%)
Gender of the child		
Female	1,445 (48%)	2,085 (50%)
Male	1,589 (52%)	2,127 (50%)
Age of the child (years)		
1	836 (28%)	1,067 (25%)
2	696 (23%)	968 (23%)
3	751 (25%)	1,115 (26%)
4	751 (25%)	1,062 (25%)
Economic variable		
Wealth quintile		
Middle	684 (23%)	922 (22%)
Lower	571 (19%)	848 (20%)
Lowest	555 (18%)	755 (18%)
Richer	631 (21%)	912 (22%)
Highest	593 (20%)	774 (18%)
Obstetric variables		
Planned current pregnancy		
Yes	2,162 (71%)	3,183 (76%)
No	872 (29%)	1029 (24%)
Moment of the 1st CPN		
First quarter	1,234 (41%)	1,411 (33%)
Second quarter	1,325 (44%)	2,342 (56%)
Third quarter	475 (16%)	459 (11%)
ANCs visits number		
0–3	1,236 (41%)	2,568 (61%)
≥ 4	1,798 (59%)	1,644 (39%)
Composite coverage index (CCI) variables		
At least one antenatal care visit with a qualified provider		
Yes	2,238 (74%)	3,522 (84%)
No	796 (26%)	690 (16%)
Childbirth assisted by qualified personnel		
Yes	1,395 (46%)	2,604 (62%)
No	1,639 (54%)	1,608 (38%)
Family planning request satisfied		
Yes	270 (8.9%)	656 (16%)
No	2,764 (91.1%)	3556 (84%)
Pentavalent		
Yes	1,714 (56%)	1,700 (40%)
No	1,320 (44%)	2,512 (60%)
Vaccination against Measles		
Yes	2,188 (72%)	1,912 (45%)
No	846 (28%)	2,300 (55%)
Vaccination against BCG		
Yes	2,702 (89%)	2,840 (67%)
No	332 (11%)	1372 (33%)
Oral rehydration therapy for children with diarrhoea		
Yes	1,826 (60%)	3,360 (80%)
No	1,208 (40%)	852 (40%)
Seeking care for pneumonia		
Yes	2,272 (75%)	2,597 (62%)
No	762 (25%)	1,615 (38%)

**Table 2:** CCI of women and their children in 2012 and 2018

CCI	DHS 2012	DHS 2018
Partial	1,897 (62.5%)	1,661 (39%)
Optimal	1,137 (37.5%)	2,551 (61%)

### Bivariate analysis

The following variables were significantly associated with the CCI: wealth quintiles, marital status, mother's education, access to the media, number of living children, planned pregnancy in progress, education of the partner, time of completion of the ANCs and the number of ANCs (Table 3). In 2018 the previous variables were significantly associated with CCI except for the variable planned pregnancy in progress (Table 3).

**Table 3:** Univariate analysis between coverage and characteristics of women aged 15–49 years and children under five years in Guinea. DHS 2012 and 2018

	DHS 2012			DHS 2018		
Characteristics	Optimal coverage *N* = 1,137	Partial coverage *N* = 1,897	*p*-value^1^	Partial coverage *N* = 1,661	Optimal coverage *N* = 2,551	*p*-value^1^
Sociodemographic variables						
Age of mothers			0.3			0.070
15–18	60 (5.3%)	106 (5.6%)		68 (4.1%)	117 (4.6%)	
19–30	670 (59%)	1,147 (60%)		919 (55%)	1,489 (58%)	
31t-40	327 (29%)	552 (29%)		541 (33%)	794 (31%)	
41–49	80 (7.0%)	92 (4.8%)		132 (8.0%)	152 (6.0%)	
Wealth quintile			< 0.001			< 0.001
Highest	52 (4.6%)	541 (29%)		138 (8.3%)	636 (25%)	
Middle	272 (24%)	412 (22%)		391 (24%)	532 (21%)	
Lower	257 (23%)	314 (17%)		429 (26%)	419 (16%)	
Lowest	367 (32%)	188 (9.9%)		487 (29%)	268 (11%)	
Richer	189 (17%)	443 (23%)		215 (13%)	696 (27%)	
Marital status			< 0.001			< 0.001
Married	1,076 (95%)	1,702 (90%)		1,585 (95%)	2,362 (93%)	
Single	61 (5.3%)	196 (10%)		76 (4.6%)	189 (7.4%)	
Number of household members			0.2			0.6
1–5	304 (27%)	553 (29%)		478 (29%)	748 (29%)	
6–10	533 (47%)	910 (48%)		883 (53%)	1,310 (51%)	
> 10	300 (26%)	434 (23%)		300 (18%)	493 (19%)	
Education of the mother			< 0.001			< 0.001
Higher	2 (0.2%)	53 (2.8%)		18 (1.1%)	81 (3.2%)	
No formal education	990 (87%)	1,262 (66%)		1,412 (85%)	1,792 (70%)	
Primary	102 (9.0%)	300 (16%)		146 (8.8%)	267 (10%)	
Secondary	42 (3.7%)	282 (15%)		86 (5.1%)	412 (16%)	
Age of the head of household			0.3			0.10
16–25	42 (3.7%)	47 (2.5%)		48 (2.9%)	82 (3.2%)	
26–40	401 (35%)	737 (39%)		624 (38%)	1,060 (42%)	
41–60	495 (44%)	775 (41%)		745 (45%)	1,092 (43%)	
> 60	199 (18%)	339 (18%)		244 (15%)	318 (12%)	
Currently working			< 0.001			0.014
Yes	964 (85%)	1,467 (77%)		1,110 (67%)	1,946 (76%)	
No	173 (15%)	430 (23%)		551 (33%)	605 (24%)	
Access to newspapers			< 0.001			< 0.001
Access	11 (1.0%)	183 (9.6%)		35 (2.1%)	256 (10%)	
No access	1,125 (99%)	1,715 (90%)		1,626 (98%)	2,295 (90%)	
Access to the radio			< 0.001			< 0.001
Access	644 (57%)	1,270 (67%)		889 (54%)	1,763 (69%)	
No access	493 (43%)	627 (33%)		772 (46%)	789 (31%)	
Access to the TV			< 0.001			< 0.001
Access	274 (24%)	969 (51%)		475 (29%)	1,417 (56%)	
No access	863 (76%)	929 (49%)		1,186 (71%)	1,134 (44%)	
Education of the mothers’ partner			< 0.001			< 0.001
Higher	24 (2.1%)	201 (11%)		46 (2.8%)	281 (11%)	
No formal education	900 (79%)	1,048 (55%)		1,327 (80%)	1,631 (64%)	
Primary	125 (11%)	282 (15%)		114 (6.8%)	194 (7.6%)	
Secondary	87 (7.7%)	367 (19%)		175 (11%)	445 (17%)	
Number of living children			< 0.001			0.001
1–2	372 (33%)	842 (44%)		567 (34%)	992 (39%)	
3–4	377 (33%)	604 (32%)		608 (37%)	949 (37%)	
5–7	336 (30%)	409 (22%)		430 (26%)	538 (21%)	
> 7	52 (4.6%)	42 (2.2%)		56 (3.4%)	73 (2.9%)	
Child gender			> 0.9			0.10
Female	540 (47%)	905 (48%)		793 (48%)	1,292 (51%)	
Male	597 (53%)	992 (52%)		868 (52%)	1,259 (49%)	
Obstetric variables						
Planned current pregnancy			< 0.001			0.10
Yes	856 (75%)	1,306 (69%)		1,231 (74%)	1,952 (77%)	
No	281 (25%)	591		430 (26%)	599 (23%)	
Moment of the 1st CPN			< 0.001			0.001
First quarter	376 (33%)	858 (45%)		481 (29%)	930 (36%)	
Second quarter	449 (40%)	876 (46%)		900 (54%)	1,442 (57%)	
Third quarter	312 (27%)	163 (8.6%)		280 (17%)	179 (7.0%)	
ANCs visits number			< 0.001			< 0.001
0–3	700 (62%)	536 (28%)		507 (84%)	1,034 (85%)	
≥ 4	437 (38%)	1,362 (72%)		96 (16%)	177 (15%)	

^1^Wilcoxon rank-sum test for complex survey samples; chi-squared test with Rao & Scott's second-order correction

### Multivariate analysis

Table 4 shows the multivariate analysis between the CCI and the various factors. In 2012, all quintile classes had a lower probability of having an optimal CCI than the highest quintiles. For the middle wealth quintile OR = 0.29 [95% CI; 0.18, 0.48], for the lower wealth quintile OR = 0.25 [95% CI; 0.15, 0.40], for the lowest wealth quintile OR = 0.11 [95% CI; 0.07, 0.18], for the higher wealth quintile OR = 0.32 [95% CI; 0.21, 0.51]. Women who started their ANCs in the second half of the year were 60% more likely to have optimal CCI than women who started their ANCs in the first quarter. Those who had done 4 ANCs were 2.78 times more likely to have an optimal CCI than those with less OR = 2.78 [2.24, 3.45]. In 2018, three of four wealth quintiles had a lower probability of having an optimal CCI than the highest quintiles. For middle OR = 0.60 [95% CI; 0.43, 0.83], for poor OR = 0.45 [95% CI; 0.32, 0.64], for lowest OR = 0.27 [95% CI; 0.19, 0.38]. Women who did not have access to newspapers were 46% less likely to have optimal CCI than those who had access to them. Women with planned pregnancies were 28% more likely to have an optimal CCI than those without planned OR = 1.28 [95% CI;1.05, 1.56]. All education levels of the partner who were below the university level had a lower probability of having an optimal CCI than partners who had a university level. Women with more than 4 ANCs were 2.43 times more likely to have an optimal CCI than those with the least OR = 2.43 [95% CI;2.03, 2.90]. Finally, those who worked were 87% more likely to have an optimal BMI than those without OR = 1.87 [95% CI;1.55, 2.25].

**Table 4:** Multivariate analysis between CCI and characteristics of women aged 15–49 and children under 5 in Guinea. DHS MICS 2012 and 2018

	DHS 2012			DHS 2018		
Characteristics	AOR^*1*^	95% CI^*1*^	*p*-value	AOR^*1*^	95% CI^*1*^	*p*-value
Wealth quintile			< 0.001			< 0.001
Richest	—	—		—	—	
Middle	0.29	0.18, 0.48	< 0.001	0.60	0.43, 0.83	0.003
Poorer	0.25	0.15, 0.40	< 0.001	0.45	0.32, 0.64	< 0.001
Poorest	0.11	0.07, 0.18	< 0.001	0.27	0.19, 0.38	< 0.001
Richer	0.32	0.21, 0.51	< 0.001	1.05	0.78, 1.41	0.7
Sex of the head of the household						0.016
Female	—	—		—	—	
Male	0.81	0.59, 1.12	0.2	1.19	0.90, 1.58	0.070
Mother’s level of education						
Higher	—	—		—	—	
No education	0.44	0.10, 1.98	0.3	1.71	0.74, 3.95	0.2
Primary	0.58	0.13, 2.63	0.5	1.49	0.65, 3.42	0.3
Secondary	0.69	0.16, 2.95	0.6	2.30	0.98, 5.44	0.057
Marital status						
Married				—	—	
Single				1.32	0.90, 1.95	0.2
Age of the head of household						
[16–25]	—	—		—	—	
[26–40]	1.47	0.80, 2.70	0.2	0.82	0.46, 1.46	0.5
[41–60]	1.15	0.62, 2.15	0.7	0.74	0.41, 1.33	0.3
[61 and more]	1.10	0.58, 2.09	0.8	0.59	0.33, 1.07	0.083
Access to newspapers						
Access	—	—		—	—	
No access	0.54	0.23, 1.30	0.2	0.54	0.35, 0.84	0.006
Number of living children						
[1, 2]	—	—			—	
[3, 4]	0.97	0.77, 1.22	0.8			
[5–7]	0.80	0.62, 1.04	0.10			
[7 and more]	0.57	0.30, 1.08	0.084			
Grossesse en cours planifiée						
No	—	—		—	—	
Yes	0.82	0.65, 1.03	0.090	1.28	1.05, 1.56	0.013
Education level of the mothers’ partner						
Higher	—	—		—	—	
No education	0.66	0.35, 1.26	0.2	0.53	0.35, 0.80	0.002
Primary	0.88	0.44, 1.76	0.7	0.56	0.33, 0.97	0.037
Secondary	1.07	0.53, 2.18	0.8	0.58	0.38, 0.88	0.011
Moment of the 1st ANC						
First quarter	—	—		—	—	
Second quarter	1.60	1.28, 2.01	< 0.001	1.17	0.97, 1.41	0.092
Third quarter	0.90	0.67, 1.22	0.5	0.67	0.51, 0.89	0.005
ANCs visits number						
[0–3]	—	—		—	—	
[4 and more]	2.78	2.24, 3.45	< 0.001	2.43	2.03, 2.90	< 0.001
Gender of the child						
Female	—	—		—	—	
Male	0.81	0.59, 1.12	0.2	1.19	0.90, 1.58	0.2
Access to the radio						
Access				—	—	
No access				0.75	0.63, 0.90	0.002
Access to television						
Access				—	—	
No access				0.68	0.55, 0.84	< 0.001
Currently working						
No				—	—	
Yes				1.87	1.55, 2.25	< 0.001

^1^*OR* Odds Ratio, *CI* Confidence Interval

### Classification and the regression tree

**Fig. 2:**
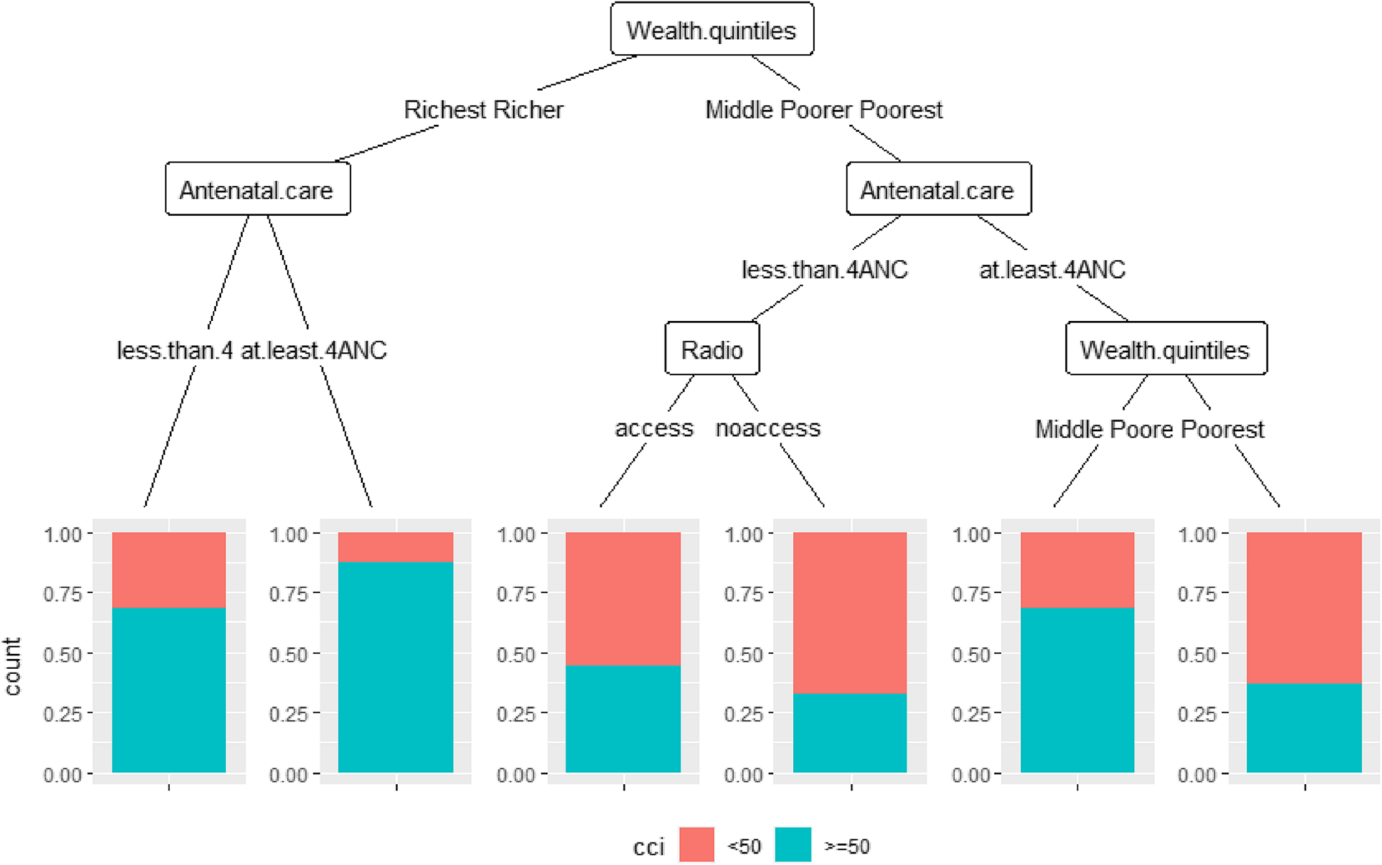
Classification and regression tree for CCI and sociodemographic and economic characteristics. DSH 2012

**Fig. 3:**
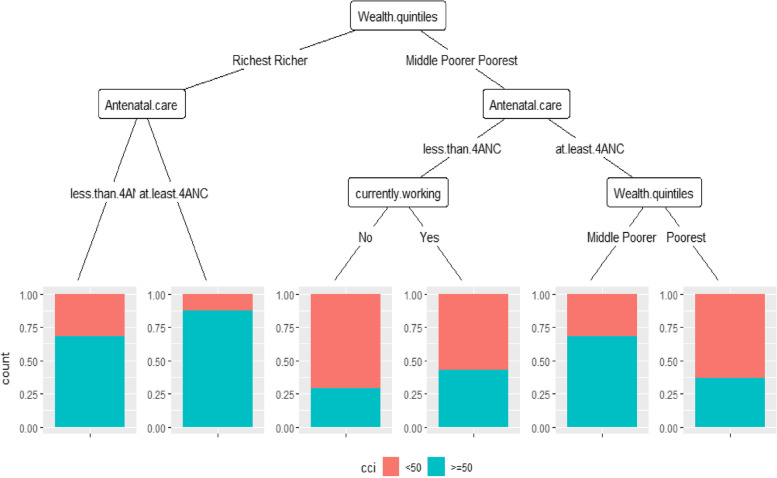
Classification and regression tree for CCI and sociodemographic and economic characteristics. DSH 2018

Figure 2 shows the regression tree for the 2012 DHS. Women from the prosperous and wealthy class with at least 4 ANCs had a higher optimal CCI than those with less. According to the wealth quintiles, women who were in the following quintiles (low and very low) had a lower optimal CCI than those in the middle quintile. Finally, women who had access to the radio had a higher optimal CCI compared to those who did not have access to it. Figure 3 shows the regression tree for the 2018 DHS. Among women in the higher and highest quintiles, those who had done at least 4 ANCs had a higher optimal CCI. Among those who had less than 4 ANCs, those who worked had a higher optimal CCI than those who did not. Finally, women in the middle quintile had a higher CCI than those in the poor and poorer quintile.

### Spatial analysis

**Fig. 4:**
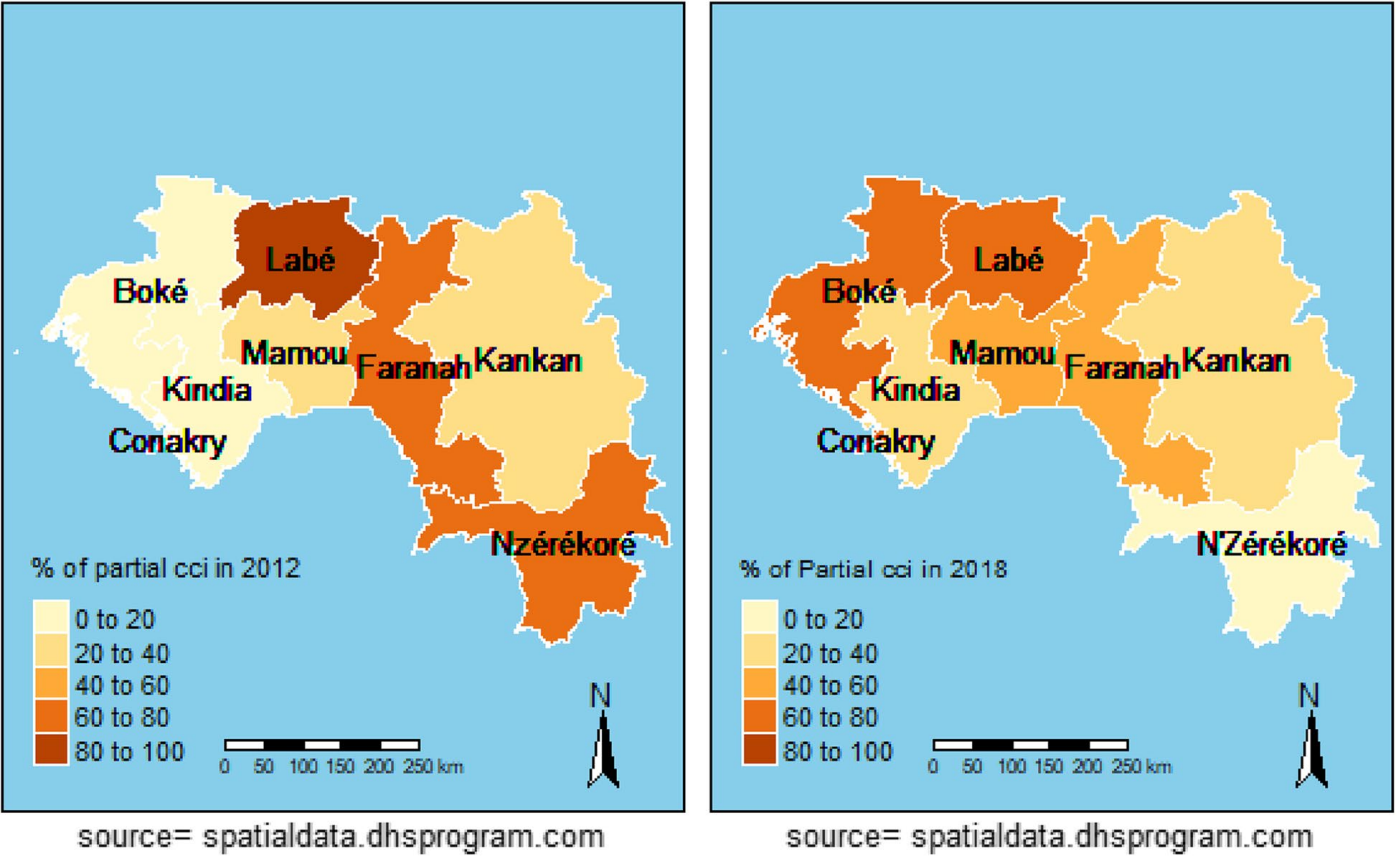
Maps representation of the CCI according to administrative regions for DHS 2012 and 2018

**Fig. 5:**
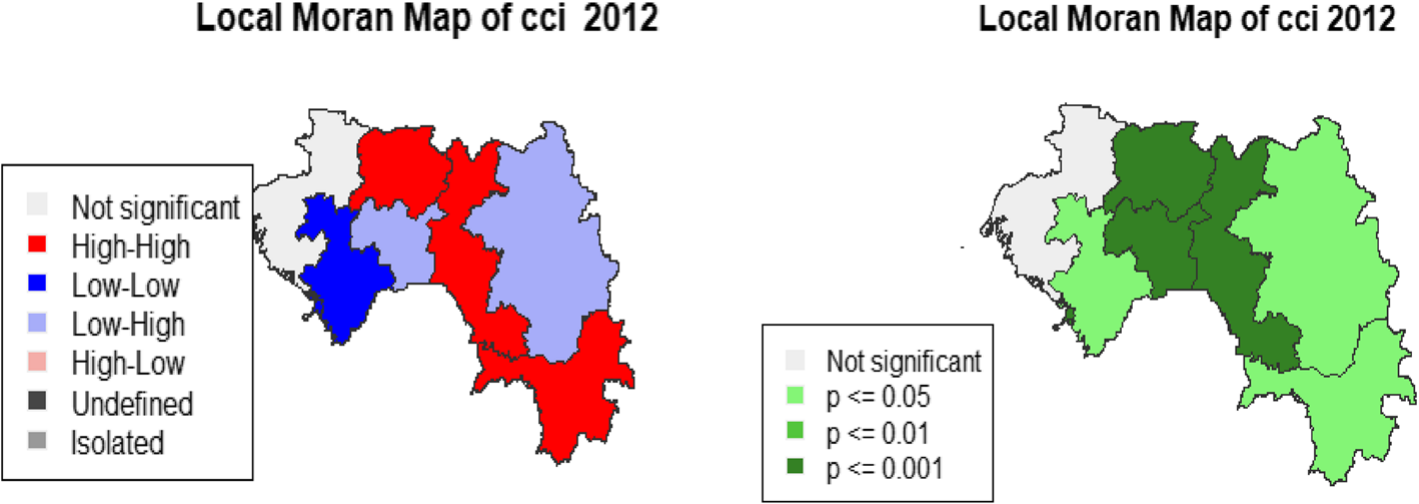
Maps of the CCI’s local spatial association indicators (LISA) according to administrative regions

**Fig. 6:**
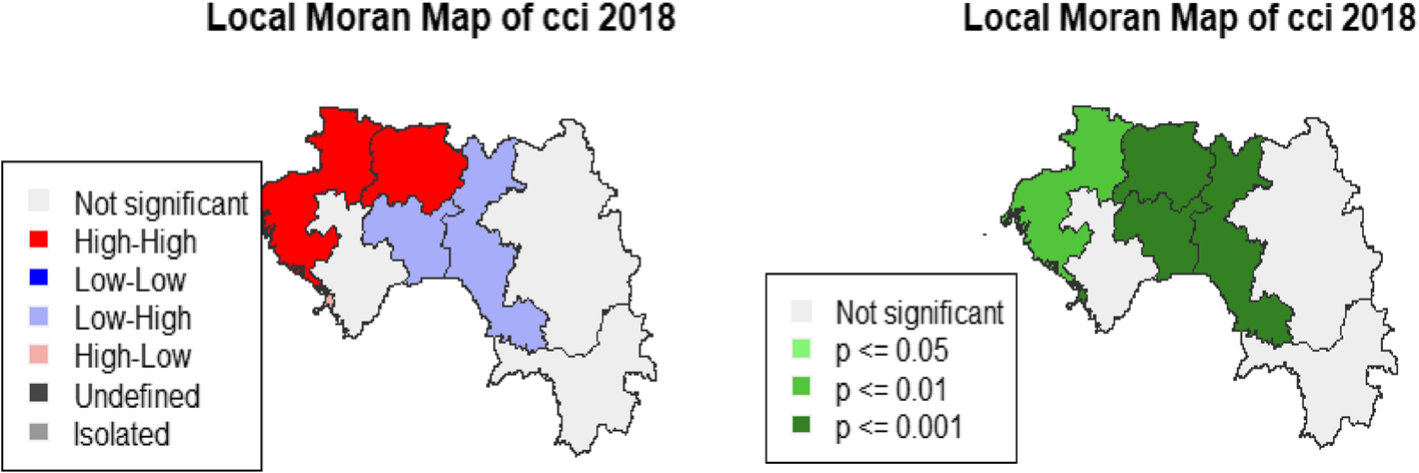
Maps of the CCI's local spatial association indicators (LISA) according to administrative regions

DHS 2012 revealed the highest partial CCI coverage in Labé, Faranah and N'zérokoré. In 2018, the Conakry, Boké and Labé regions recorded a higher partial CCI than others (Fig. 4). Table 5 shows the analysis of the global autocorrelation. In 2012 and 2018, the global Moran Index (GMI) indicates that there is a significant association (GMI = 0.09, p < 0.01 and IGM = 0.07, p < 0.01, respectively). In 2012, LISA's analysis showed a statistically significant autocorrelation, with aggregates of low–high negative values in Kankan and Mamou and also aggregated of high-high positive values in Faranah, Labé and N'zérékoré, low-low in Kindia and Conakry (Fig. 5). In 2018, LISA's analysis showed a statistically significant autocorrelation, with aggregates of negative values in Faranah, Mamou (weak-strong) and Conakry (strong–weak); and also aggregates of strong-strong positive values in Faranah and Labé (Fig. 6).

**Table 5:** Global Moran Index for DHS 2012 and 2018

DHS	Global moran index	*p*-value
DHS 2012	0.09	< 0.01
DHS 2018	0.07	< 0.01

## Discussions

The maternal and child health analysis using the CCI showed that optimal coverage increased markedly from 2012 to 2018. That result indicates the progress made by the government to improve interventions for women and children. Our results suggest that the highest wealth quintiles households had more access to interventions with a greater probability of having a higher optimal CCI. These observations are in line with other studies, which indicate the rich are ahead of the poor in accessing a higher CCI, for example, in the context of the evaluation of nutrition among children in sub-Saharan Africa [23], in the context of reproductive health [24] and the empowerment of women [12]. Similarly, the results reveal that women who started their ANCs in the year's second half are likelier to have an optimal CCI. Although surprising, the outcome could be the decrease in the intensity of visits from this period. Indeed, one can hypothesise that the more visits to the health centres there are, the fewer women move to follow them in full; in addition, most of these women belong to the middle class or the low-wealth quintile. In addition, there is also the fact that wealthy women with the opportunities to take care of themselves most often prefer to consult private structures and, secondarily, public ones. Also, some women start ANCs in one place and continue elsewhere without documentation that can trace the course of care. Through these observations, we understand why the women we met in the year's second half do not necessarily have poor health results.

A continuation of the previously associated factor, the number of ANCs is an essential indicator of a good follow-up of the lump, especially for women who do not have the opportunities to consult elsewhere, such as in private structures. During these ANCs, the emphasis is also on post-natal follow-up. Access to information, mainly that of newspapers, is a factor in improving the CCI. Messages through newspapers and the media are essential in improving health use. By way of example, Bababola et al. A positive association between media communication and family planning [25]. The effect of media communication on health is sufficiently documented [26–28]. The educational level of the partners is also associated with the CCI. Indeed, in most African societies and Guinea, health decisions are made by the head of the household or the husband of women. Therefore, a high level of education for the spouse would facilitate the search for the appropriate information. This result is similar to Tekelab T et al.'s study on factors affecting ANCs in Ethiopia [29]. In 2018, women whose current pregnancies were more likely to have an optimal CCI than those who had not planned it. We apprehended the previous situation from different perspectives. If we take the example of unwanted pregnancies, it is evident that in Guinean culture, these women benefit from less attention from the family; the fundamental reason is the prohibition of pregnancies out of wedlock in most communities in Guinea.

Moreover, if the woman is married, the decision to get pregnant does not depend on her in most cases, leading to low use of services, especially in poverty. The positive association between pregnancy planning and using ANCs has also been reported in Ethiopia [29]. In addition, we also note that working women had a higher chance of having an optimal CCI. This result aligns with what we have highlighted above; working women have a certain autonomy that allows them to afford the best care for themselves and their children. Although the care is theoretically free, access to this care can be an obstacle, especially for those who do not work. We cannot, for example, the cost of travel. Indeed, for some women, the proximity of health structures is an advantage; however, many are far from it. Therefore, they must take transportation at an often-unaffordable price.

Spatial analysis indicates that the highest proportions of partial CCI were in Labé, Faranah and N'zérokoré in 2012 and Boké, Conakry and Labé in 2018. This result suggests that efforts lessened in the Boké and Conakry regions, which registered a small proportion of partial CCI in 2012. We can point out the sociopolitical instability among the reasons explaining the latter finding. For example, the city of Conakry has suffered the full consequences of Ebola, which has shaken the health system [30]. In addition to this significant event, several political demonstrations took place between 2015 and 2018; these situations were likely to disrupt the ordinary course of activities, including the movement of people. We must emphasise that the level of vaccination of children in Conakry remains low compared to expectations. The Labé region, on the other hand, has made slight progress in reducing partial CCI but remains among the areas of high proportions. The region's geographical location (presence of rugged reliefs) making access difficult somewhat explains this situation. Local spatial autocorrelation indicates that the Faranah region and Labé have high partial CCI values.

Regarding the Faranah region, there are fewer health interventions (external support) compared to other parts. Being a remote area from the capital, considering accessibility, the staff of caregivers assigned there most often do not design to stay there, which can affect the quality of services.

### Strength and limitation

DHS study is nationwide; therefore stands for good data for inference. The study's retrospective nature does not allow further exploration than what we have. We only used two DHS surveys instead of all DHS available. We opted as such to select the most recent surveys describing the era of many political and economic changes. The transformation of the CCI variable in binary leads to information losses but shows overall maternal and child health progress. Some information regarding the indicators of vaccination relied on the mothers' statements could induce the risk of memorisation. The unit of the spatial scale is the region; for a more refined analysis, it would have been interesting to integrate the district level to inform local policies.

Nevertheless, this study was aimed only at the regional level for decision-makers at the national level. We did not study other factors, such as breakdowns, the quality of service and the perception of beneficiaries. The absence of these elements limited us from going deeper. Finally, the scheme of the study is limited to concluding causality.

## Conclusion

This study reveals substantial improvements between the two DHS regarding the optimal CCI. These improvements should be continued and strengthened. One of the crucial factors found is prenatal follow-up. It is essential to think about the mechanisms for raising the level of use of antenatal care visits. Also, policies should implement strategies to reduce inequalities between rich and poor women. One of the strategies could consist in improving access to information on the CCI indicators by recalling using new information technologies (self-generated telephone messages, Facebook) [27] and community media (days of broadcasts coupled with phone calls or interactions with the public) [28]. In addition, it is vital to bring care closer to people with low incomes and ease access to areas such as the Labé. Finally, evaluating the efficiency of these different strategies is necessary from a sustainability perspective.

## Supplementary Information

**Additional file 1.**

## Data Availability

The datasets analysed during the study were obtained with permission from ICF International and are available at https://dhsprogram.com/data/dataset/Guinea. The R script used for the analysis is included in the supplementary file section.) accordingly.

## Abbreviations

ANCs: Antenatal care visits
AIC: Aikake information criterion
AOR: Adjusted odds ratio
BCG: Baccille calmatte guérin
CART: Classification and regression trees
CCI: Composite coverage index
CI: Confidence interval
CPNM: Care seeking for pneumonia
DHS: Demographic and health survey
FPS: Family planning satisfied
GMI: Global moran index
ICF: Inner city fund
MSL: Measle
ORT: Oral rehydration therapy
SBA: Skill birth attendant
WHO: World health organization
