# Supplementary material for: Evaluation of maternal and child care continuum in Guinea: a secondary analysis of two demographic and health surveys using the composite coverage index (CCI)
Source: BMC Pregnancy Childbirth. 2023 May 27;23:391. doi: 10.1186/s12884-023-05718-y (PMC10224326; doi:10.1186/s12884-023-05718-y)
Supplement: Supplementary file 1 — Additional file 1. [file 12884_2023_5718_MOESM1_ESM.pdf]

Analysis of maternal and child health indicators: the composite coverage index (CCI) used in Guinea from 2012 to 2018.

Authors' affiliation

Diao CISSE[1,9]*, Almamy Amara TOURE*[,¥,1,2], Abdourahamane DIALLO[3], Juste Aristite GOUNGOUNGA[4,5], Jean Jaques Olivier KADIO[1,6], Ibrahima BARRY[2], Souleymane BERETE[7], Aboubacar Sidiki MAGASSOUBA[1], Souleymane Hassane HAROUNA[8], Alseny Yarie CAMARA[2], Younoussa SYLLA[2], Kola CISSE[9], Maïmouna SIDIBE[10], Abdoulaye TOURE[6], Alexandre DELAMOU[1,2,11].

* These authors contributed equally to this work.

1 Department of Public Health, Faculty of Health Sciences and Techniques, Gamal Abdel Nasser University, Conakry, Guinea. 2 Centre National de Formation et Recherche en Santé Rurale de Maférinyah.3 Centre Hospitalo-Universitaire Ignace Deen, Service de Gynécologie, Conakry, Guinée. 4 Université de Bourgogne, Registre Bourguignon des cancers digestifs, Dijon, France.5 Inserm UMR1231, UMR1231, Dijon, France.6 Centre de Recherche et de Formation en Infectiologie de Guinée.7 Catholic Relief Service .8 Action Damien Guinée.9 Médecins Sans Frontière Espagne, Mali.10 Centre Hospitalo-Universitaire Fann, Service de Maladies Infectieuses

[¥] **Corresponding author**

Almamy Amara TOURE, Centre National de Formation et de Recherche en Santé Rurale de Maferinyah, Forecariah, Guinea, almamy@maferinyah.org

et Tropicales, Dakar, Sénégal. 11 Centre d´Excellence Africain pour la Prévention et le Contrôle des Maladies Transmissibles (CEA-PCMT), Gamal Abdel Nasser University, Guinea

Address's email of the authors

Diao CISSE : diaosg49@gmail.com

Almamy Amara TOURE : almamy@maferinyah.org

Abdourahmane DIALLO : adiallo69gn@yahoo.fr

Juste Aristite GOUNGOUNGA : goungoungajuste@gmail.com

Kadio Jean Jaques Olivier KADIO : drkadiojolivier@gmail.com

Ibrahima BARRY: barryibra7@gmail.com

Souleymane BERETE : sberete48@gmail.com

Aboubacar Sidiki MAGASSOUBA : magasbakary01@yahoo.fr

Souleymane Hassane HAROUNA : hassoul20@yahoo.fr

Alseny Yarie CAMARA : camaraall35@gmail.com

Younoussa SYLLA : doctorsyllayounoussa@gmail.com

Kola CISSE : cissekola3@gmail.com

Maïmouna SIDIBE : maisidibe.kl@gmail.com

Abdoulaye TOURE : abdoulayek2002@yahoo.fr

Alexandre DELAMOU : adelamou@maferinyah.org

```
library(foreign)

kr12<- read.spss("D:\\MOOC\\GNKR62FL.sav",to.data.frame = TRUE)

kr12 <- subset(kr12, select = c("V001", "V005", "V007", "V012", "V013", "V021",
                    "V022","V023", "V024", "V025", "V026", "V130", "V131", "V136", "V149","V151",
                            "V152", "V157", "V158", "V159", "V190","V191" ,"V218", "V225",
        "V501","V502","V701",
                    "V714", "V626", "V313",

"B4","B5","B8","MIDX","H1","H2","H3","H5","H7","H9","H10","H11","H13","H14",
                    "H31","H32A","H32B","H32C","H32E","H32I","H32J","H32K","H32L", "H32N",
"H32S","H32Y",
                    "M2A","M2B","M2C","M3A","M3B","M3C", "M13", "M14"))




#Filtrer les enfants vivants

library(nlme)

library(dplyr)

##################################################################Filtering on living child

krv12 <- filter(kr12,kr12$B5=="Yes")

#########################################################Filtering by age more than a year

krv12 <- filter(kr12,kr12$B8>=1)
```

```
##################################################checking for missing values
sapply(krv12, function(x) sum(is.na(x)))
##########################################Recoding variables before imputation
summary(krv12$V012)
table(krv12$V012)

##Recoding for living child and household number
summary(krv12$`V218`)
table(krv12$`V218`)
summary(krv12$`V136`)
table(krv12$`V136`)

str(krv12)
summary(krv12)
sapply(krv12, function(x) sum(is.na(x)))

###################################################Reducing data sets
##############recoding some variables
levels(krv12$H2)
table(krv12$H2)
levels(krv12$H2) <- c("No", "Yes","Yes", "Yes", "No")
levels(krv12$H2)

table(krv12$H2)

levels(krv12$H3)
table(krv12$H3)
levels(krv12$H3) <- c("No", "Yes","Yes", "Yes", "No")
levels(krv12$H3)
```

```
levels(krv12$H5)
table(krv12$H5)
levels(krv12$H5) <- c("No", "Yes","Yes", "Yes", "No")
levels(krv12$H5)
table(krv12$H5)


levels(krv12$H7)
table(krv12$H7)
levels(krv12$H7) <- c("No", "Yes","Yes", "Yes", "No")
levels(krv12$H7)
table(krv12$H7)

levels(krv12$H9)
table(krv12$H9)
levels(krv12$H9) <- c("No", "Yes","Yes", "Yes", "No")
levels(krv12$H9)
table(krv12$H9)


levels(krv12$H10)
table(krv12$H10)
levels(krv12$H10) <- c("No", "Yes", "No")
table(krv12$H10)

levels(krv12$H11)
table(krv12$H11)
levels(krv12$H11) <- c("No", "Yes", "Yes", "No")
table(krv12$H11)
```

```
levels(krv12$H13)
table(krv12$H13)
levels(krv12$H13) <- c("No", "Yes", "Yes", "No")
table(krv12$H13)

levels(krv12$H14)
table(krv12$H14)
levels(krv12$H14) <- c("No", "Yes", "Yes", "No")
table(krv12$H14)

levels(krv12$H31)
levels(krv12$H31) <- c("No", "Yes", "Yes", "No")
table(krv12$H31)

#########################Information access
levels(krv12$V157)
levels(krv12$V157) <- c("noaccess", "access", "access", "access")
table(krv12$V157)

levels(krv12$V158)
levels(krv12$V158) <- c("noaccess", "access", "access", "access")
table(krv12$V158)

levels(krv12$V159)
levels(krv12$V159) <- c("noaccess", "access", "access", "access")
table(krv12$V159)
levels(krv12$V502)
table(krv12$V502)
levels(krv12$V502) <-  c("single","married","single")
```

```
table(krv12$V502)


levels(krv12$V149)
levels(krv12$V149) <- c("noeducation", "primary", "primary", "secondary",
                        "secondary", "higher")
table(krv12$V149)

##########################################partner education
levels(krv12$V701)
levels(krv12$V701) <- c("noeducation","primary","secondary","higher","noeducation")
table(krv12$V701)



levels(krv12$`V152`)
levels(krv12$`V152`) <- c("18","19","20","21","22","23","24","25","26","27",
                          "28","29","30","31","32","33","34","35","36","37","38","39",
                          "40","41","42","43","44","45","46","47","48","49","50","51",
                          "52","53","54","55","56","57","58","59","60","61","62","63",
                          "64","65","66","67","68","69","70","71","72","73","74","75",
                          "76","77","78","79","80","81","82","83","84","85","86","87",
                          "88","89","90","91","92","95","95","95")
krv12$`V152`<- as.numeric(levels(krv12$`V152`))[krv12$`V152`]
table(krv12$`V152`)
summary(krv12$`V152`)
table(krv12$`V152`)

##########################################anc
table(krv12$`M14`)
levels(krv12$`M14`)
```

```
levels(krv12$`M14`) <-
c("0","1","10","11","12","13","14","15","16","17","18","2","20","3","4","5","6","7","8","9","")

table(krv12$`M14`)

krv12$`M14` <- as.numeric(levels(krv12$`M14`)[krv12$`M14`])

table(krv12$`M14`)

##############################

summary(krv12$`M14`)

#krv12$`M14` <- cut(krv12$`M14`,breaks = c(0,1,3,9),include.lowest = TRUE)

table(krv12$`M14`)

##########################################time first anc

levels(krv12$`M13`)

table(krv12$`M13`)

levels(krv12$`M13`) <- c("0","1","2","3","4","5","6","7","8","9","0")

table(krv12$`M13`)

krv12$`M13` <- as.numeric(levels(krv12$`M13`)[krv12$`M13`])

table(krv12$`M13`)


summary(krv12$`M13`)

#krv12$`M13` <- cut(krv12$`M13`,breaks = c(1,3,6,9),include.lowest = TRUE, labels = c("first-
trim","second-trim","third-trim"))

table(krv12$`M13`)


#ORT#####################################################For diarrhea

krv12$ORT[krv12$H11=="Yes" & krv12$H13=="No" &krv12$H14=="No"] <- "0"

krv12$ORT[krv12$H11=="Yes" & krv12$H13=="Yes" |krv12$H14=="Yes"] <-"1"


levels(krv12$ORT)

krv12$ORT <- factor(krv12$ORT)

table(krv12$ORT)

krv12$ORT <- factor((krv12$ORT),levels = c("0","1"))

krv12$ORT<-as.numeric(levels(krv12$ORT)[krv12$ORT])
```

```
table(krv12$ORT)


#####################################CPNM is care seeking for pneumonia

krv12$CPNM[krv12$H31=="Yes" &
        krv12$H32A=="No" &
        krv12$H32B=="No" &
        krv12$H32C=="No" &
        krv12$H32E=="No" &
        krv12$H32J=="No" &
        krv12$H32L=="No" &
        krv12$H32N=="No" &
        krv12$H32S=="No" ] <-
  "0"
krv12$CPNM[krv12$H31=="Yes" &
        krv12$H32A=="Yes" |
        krv12$H32B=="Yes" |
        krv12$H32C=="Yes" |
        krv12$H32E=="Yes" |
        krv12$H32J=="Yes" |
        krv12$H32L=="Yes" |
        krv12$H32N=="Yes" |
        krv12$H32S=="Yes" ] <-
  "1"
krv12$CPNM <- factor(krv12$CPNM)
sapply(krv12, function(x) sum(is.na(x)))

###############################Removing uninteristing variables for imputation
krv12d <- krv12[-c(1:3,5,7:13,22,25:26,29:30,32,34:35,42:55)]
```

```
krv12d1 <- krv12[c(1:3,6:7,9:10,12:13,21:22,26,29,34)]
####################################Vaccination  status
library(mice)
init = mice(krv12d, maxit=0)
#init = mice(krv18d, maxit=20)

meth1 = init$method
#meth1[which(meth1=="pmm")]<-"norm.boot"
#meth1[which(meth1=="logreg")]<-"logreg.boot"

meth1
predM = init$predictorMatrix
#predM[, c("V001")] <- 0
#predM[, c("V005")] <- 0
#predM[, c("V191")] <- 0
krv12d = mice(krv12d,pred=predM,seed = 15422,m = 5)
#krv18d = mice(krv18d,pred=predM,seed = 15422,m = 10)
#imp1=mice(krv18d,print=FALSE)
##################################Inspect the convergence of the algorithm
plot(krv12d,layout = c(4,4))#####based on fig we need more iterations
#########################Inspect diagnostics
densityplot(krv12d)
densityplot(krv12d, ~ CPNM+M13+M14,
        layout = c(3,2))
bwplot(krv12d)
krv12d <- complete(krv12d)
##########################################################saving workspace
#save.image(file =("D:\\MOOC\\diao1.RData"))
#########################################################Loading workspace
#load("D:\\MOOC\\diao1.RData")
```

```
str(krv12d)
sapply(krv12d, function(x) sum(is.na(x)))
summary(krv12d)
#################################################################Merging dataframes
diaoc <- cbind(krv12d1,krv12d)
sapply(diaoc, function(x) sum(is.na(x)))

#############################################Following code not check!!!!!!!!!!!!!
#write.csv2(diaoc,file = "D:\\MOOC\\diaod1.csv")

krv12 <- read.csv2("D:\\MOOC\\diaod1.csv", header = T,
          stringsAsFactors=TRUE)
library(dplyr)
################################################Including missing variables
#krv18 <- cbind(krv18,krv18d1[c(13)])
str(krv12)
summary(krv12)
sapply(krv12, function(x) sum(is.na(x)))

###########################################religion
library(na.tools)
levels(krv12$V130)
levels(krv12$V130) <- c("noreligion","Christian","muslim","noreligion")
krv12$V130 <- na.bootstrap(krv12$V130)
table(krv12$V130)

levels(krv12$V131)
levels(krv12$V131) <- c("guerzÃ©","kissi","malinkÃ©","other","peulh","soussou","toma")
table(krv12$V131)
```

```
##############################################Antenatal care
krv12$ANCS[krv12$M2A=="No" &krv12$M2B=="No" & krv12$M2C=="No"] <- "0"
krv12$ANCS[krv12$M2A=="Yes" | krv12$M2B=="Yes" | krv12$M2C=="Yes"] <- "1"
table(krv12$ANCS)

table(krv12$ANCS)
levels(krv12$ANCS)
krv12$ANCS <- factor(krv12$ANCS)
krv12$ANCS <- factor((krv12$ANCS),levels = c("0","1"))
krv12$ANCS<-as.numeric(levels(krv12$ANCS)[krv12$ANCS])
table(krv12$ANCS)

##########################################Delivery/
krv12$SBA[krv12$M3A=="No" & krv12$M3B=="No" & krv12$M3C=="No"] <- "0"
krv12$SBA[krv12$M3A=="Yes" | krv12$M3B=="Yes" | krv12$M3C=="Yes"] <-"1"
table(krv12$SBA)
krv12$SBA <- factor(krv12$SBA)
levels(krv12$SBA)
krv12$SBA <- factor((krv12$SBA),levels = c("0","1"))
krv12$SBA<-as.numeric(levels(krv12$SBA)[krv12$SBA])
krv12$SBA<-as.numeric(krv12$SBA)
table(krv12$SBA)

summary(krv12$V012)
krv12$V012 <- cut(krv12$V012,breaks = c(15,18,30,40,49),include.lowest = TRUE)
summary(krv12$V218)
krv12$V218 <- cut(krv12$V218,breaks = c(1,2,4,7,12),include.lowest = TRUE)
summary(krv12$V136)
krv12$V136 <- cut(krv12$V136,breaks = c(1,5,10,42),include.lowest = TRUE)
summary(krv12$V152)
```

```
krv12$V152<- cut(krv12$V152,breaks = c(16,25,40,60,95),include.lowest = TRUE)


library(forcats)
levels(krv12$V626)
krv12$V626 <- fct_recode(krv12$V626,
                NULL = "Never had sex",
                "No"= "Unmet need for spacing",
                "No" = "Unmet need for limiting",
                "Yes"= "Using for spacing",
                "Yes"= "Using for limiting",
                NULL = "Spacing failure",
                NULL = "Limiting failure",
                NULL = "No unmet need",
                NULL = "Not married and no sex in last 30 days",
                NULL = "Infecund, menopausal")
krv12$V626 <- na.replace(krv12$V626,.na = "No")


levels(krv12$V626)
table(krv12$V626)
##################################################Family
#krv12$FPS <- NA
krv12$FPS[krv12$V626=="No"] <- "0"
krv12$FPS[krv12$V626=="Yes"] <- "1"


levels(krv12$FPS)
krv12$FPS <- factor(krv12$FPS)
table(krv12$FPS)
krv12$FPS <- factor((krv12$FPS),levels = c("0","1"))
krv12$FPS<-as.numeric(levels(krv12$FPS)[krv12$FPS])
table(krv12$FPS)
```

```
#Trois doses DTP
#krv12$DPT3 <- NA
krv12$DPT3[krv12$H3=="No" |krv12$H5=="No" |krv12$H7=="No"] <-"0"
krv12$DPT3[krv12$H3=="Yes" &krv12$H5=="Yes" & krv12$H7=="Yes"] <-"1"
levels(krv12$DPT3)
krv12$DPT3 <- factor(krv12$DPT3)
table(krv12$DPT3)
krv12$DPT3 <- factor((krv12$DPT3),levels = c("0","1"))
krv12$DPT3<-as.numeric(levels(krv12$DPT3)[krv12$DPT3])
table(krv12$DPT3)

#Rougole
#krv12dddd$MSL <- NA
krv12$MSL[krv12$H9=="No"] <- "0"
krv12$MSL[krv12$H9=="Yes"] <- "1"

levels(krv12$MSL)
krv12$MSL <- factor(krv12$MSL)
table(krv12$MSL)
krv12$MSL <- factor((krv12$MSL),levels = c("0","1"))
krv12$MSL<-as.numeric(levels(krv12$MSL)[krv12$MSL])
table(krv12$MSL)

#BCG
#krv12dddd$BCG <- krv12dddd$H2
#krv12dddd$BCG <- NA
krv12$BCG[krv12$H2=="No"] <-"0"
krv12$BCG[krv12$H2=="Yes"] <-"1"
table(krv12$BCG)
```

```
levels(krv12$BCG)
krv12$BCG <- factor(krv12$BCG)
table(krv12$BCG)
krv12$BCG <- factor((krv12$BCG),levels = c("0","1"))
krv12$BCG<-as.numeric(levels(krv12$BCG)[krv12$BCG])
table(krv12$BCG)




############################################Last filtering with family planning
krv12d1<- filter(krv12,krv12$FPS==0)
krv12d2<- filter(krv12,krv12$FPS==1)

####################################################Merging the two dataframes
krv12 <- rbind(krv12d1,krv12d2)

###ICC
attach(krv12)
#detach(krv18d)
#krv18d$a <- FPS
#summary(krv18d$a)
krv12$b <- (SBA+ANCS)/2
summary(krv12$b)
krv12$c <- ((2*DPT3)+MSL+BCG)/4
summary(krv12$c)
krv12$d <- (ORT+CPNM)/2
summary(krv12$d)

krv12$icc <- 0.25*(krv12$FPS+krv12$b+krv12$c+krv12$d)
```

```
krv12$icc100 <- (krv12$icc)*100

str(krv12)

summary(krv12$icc100)

##############################################Filtering for diarrhea and cough

krv12n <- filter(krv12,krv12$ORT==1&krv12$CPNM==1)

##################################################For either diarrhea or cough

krv12n1 <- filter(krv12,krv12$ORT==1|krv12$CPNM==1)

###########################################################cleaning dataframes

diao <- krv12n1[-c(1,14:15,17,25,32:44,38:44,55:57)]


######################################################Recoding before weighting

###########################Will for pregnancy

levels(diao$V225)

levels(diao$V225) <- c("no","no","yes")

table(diao$V225)


summary(diao$M13)

diao$M13 <- cut(diao$M13,breaks = c(0,3,6,9),include.lowest = TRUE,labels = c("first-trim","second-trim","third-trim"))

table(diao$M13)

summary(diao$M14)

diao$M14 <- cut(diao$M14,breaks = c(0,3,20),include.lowest = TRUE,labels=c("less.than.4ANC","at.least.4ANC"))

table(diao$M14)


diao[c(29:36)] <- lapply(diao[c(29:36)],factor)


levels(diao$ORT)

levels(diao$ORT) <- c("no","yes")

levels(diao$CPNM) <-c("no","yes")

levels(diao$ANCS) <- c("no","yes")
```

```
levels(diao$FPS)  <- c("no","yes")
levels(diao$DPT3)<- c("no","yes")
levels(diao$SBA)<- c("no","yes")
levels(diao$BCG)<- c("no","yes")
levels(diao$MSL)<- c("no","yes")
diao$V007 <- factor(diao$V007)
diao$icc <- cut(diao$icc,breaks = c(0,0.49,1),include.lowest = TRUE)
table(diao$icc)
levels(diao$icc) <- c("<50",">=50")
levels(diao$icc) <- c("partial","optimal")

#diao$icc <- relevel(diao$icc,ref =">=50" )
diao$V190 <- relevel(diao$V190,ref = "Richest")

library(questionr)
diao <- rename.variable(diao,"icc","cci")
diao <- rename.variable(diao,"V190","Wealth.quintiles")
diao <- rename.variable(diao,"M14","Antenatal.care")
diao <- rename.variable(diao,"M13","Antenatal.period")

###################### complexe design
################Descriptive statistics with weighting
library(dplyr)
library(survey)
library(party)
library(gtsummary)
library(car)
library(ggplot2)
library(GGally)
library(forestmodel)
```

```
library(Epi)
library(ggeffects)
library(ggparty)
library(intsvy)
########################################Variable Weighting
#Wt <- krv18$V005/1000000
#krv18 <-cbind(krv18,Wt)

Wt <- diao$V005/1000000
krv12 <-cbind(diao,Wt)

###################### echantillon Complexe
dw1 <-svydesign(ids=~V021,data = krv12 ,strata=~V022+V024, weights=~Wt,nest = TRUE)

#################################Descriptive summary
tab2012 <- tbl_svysummary(dw1)
tab2012

#############Excluding some variables before univariate analysis
diao1 <- diao[-c(1,3,6:7,11,26,29:36)]
names(diao1) <- c("V005", "V021", "V022", "V130", "V131", "V190", "V502", "V012",
             "V136", "V149", "V151", "V152", "V157", "V158", "V159", "V218",
             "V225", "V701", "V714", "B4", "M13", "M14", "cci", "icc100")
Wt <- diao1$V005/1000000
krv12 <-cbind(diao1,Wt)

###################### echantillon Complexe
dw1 <-svydesign(ids=~V021,data = krv12 ,strata=~V022, weights=~Wt,nest = TRUE)

############################################################"
```

```
##b)  2012
tab2012 <- tbl_svysummary(dw1,by="cci")
tab2012 <- add_p(tab2012)

tab1 <- add_p(tab2012,
        test = list(
          all_continuous() ~ "svy.t.test",
          all_categorical() ~ "svy.wald.test"
        )
)
tab1

########################Mergin 2012 and 2018
tbl_merge(tbls = list(tab2012, tab2018)) %>%
  modify_spanning_header(everything() ~ NA_character_)


####################################Only use for mapping purpose
diao1 <- diao[-c(1,3,6:7,11,26,29:36)]
names(diao1) <- c("V001","V005", "V021", "V022", "V130", "V131", "V190", "V502", "V012",
          "V136", "V149", "V151", "V152", "V157", "V158", "V159", "V218",
          "V225", "V701", "V714", "B4", "M13", "M14", "cci", "icc100")
diao1 <- factor(diao1$V001)
tab2012 <- tbl_svysummary(dw1,by="cci",statistic =  all_categorical()~"{p}%",percent = "row")

##############################################Multivariate analysis
############For 2012
dput(names(diao))
tab6 <- svyglm(cci~B4+V190+V502+V136+V151+V149+V152+V157+V158+V218+
```

```
          V225+V701+V714+M13+M14,dw1,family = quasibinomial()
)
###################Second models after taking into account collinearity
tab6 <- svyglm(icc~B4+V190+V502+V136+V151+V149+V152+V157+V158+V218+
          V225+V701+V714+M13+M14,dw1,family = quasibinomial()
)
#######################################Step AIC for choosing best model
tab7 <- step(tab6)

###############################Graphical representation of the model
ggcoef_model(tab7, exponentiate = TRUE)
forest_model(tab7)
##################Variables effects for 2012/predicted values
ggeffect(tab7,"V149")
ggeffect(tab7,"V152")
ggeffect(tab7, "V190")



#################Confusion matrix/ Quality of the model/2012
icc <- predict(tab7, type = "response", newdata =dw1[["variables"]] )
head(cci)
table(cci > 0.5, diao$icc)
#############misclassification of the model
misclas <- (352+535)/3005#####29% of misclassification

############################################################Model Evaluation
############## ROC curve
ROC(form=cci ~  B4 + V190 + V151 + V149 + V152 +V158+ V225 + V701 +
    M13 + M14,plot="ROC",data = dw1[["variables"]])
#ROC(form=icc ~B4 + V190 + V151 + V149 + V152 + V225 + V701 +
```

```
#      V714 + M13 + M14,plot="ROC",data = dw1[["variables"]])
###################################################coefficients significance
anova(tab7, test = "Chisq")
##############model assessment
with(tab7,pchisq(null.deviance-deviance,df.null-df.residual,lower.tail = F))

#############################################mulitcollinearity
vif(tab7)##########no collinearity
#############################Merging univariate and multivariate analysis
#########################################
tab7 <- tab7 %>%
  tbl_regression(exponentiate = TRUE) %>%
  add_global_p(type = "II")
tab7
####################################Using gtsummary
tab7 <- tbl_regression(tab7,exponentiate = TRUE)

########## Find why second-time women used more malprev measure than 1st
tab1 <- prop.table(table(dhs$times.1ANC,dhs$w.quitile),2)
########################################Merging Multivariate data
tbl_merge(tbls = list(tab7, tab9)) %>%
  modify_spanning_header(everything() ~ NA_character_)
##########################################################Testing interactions
#a) dhs 2012 models interactions
library(glmulti)
diao2012 <- dw1[["variables"]]
tab77 <- glmulti(cci ~ B4 + V190 + V502 + V151 + V149 + V152 + V157 + V158 + V218 +
          V225 + V714 + M13,data = diao,family=binomial())

#a) Models interaction outputs
```

```
tab77 <-svyglm(cci~1+B4+V190+V151+V149+V152+V151:B4+V149:V151+V152:B4,dw1,family =
quasibinomial())


ROC(form=icc~1+B4+V190+V151+V149+V152+V151:B4+V149:V151+V152:B4,

   plot="ROC",data = diao2012)

ggcoef_model(tab77, exponentiate = TRUE)

forest_model(tab77)

#################Confusion matrix/ Quality of the model/2012

malprev <- predict(tab77, type = "response", newdata =dw1[["variables"]] )

head(malprev)

table(malprev > 0.5, dhs2012$malprev)

##############misclassification of the model

misclas <- (64+199)/781#####33% of misclassification


########we opt for our first model because the new one perform not much



##########################b) dhs 2018 models interactions

diao <- dw1[["variables"]]

tab78 <- glmulti(cci ~ B4 + V190 + V151 + V149 + V152 + V157 + V218 + V225 + V701 +

M13 + M14,data = dhs2018,family=binomial())


#a) Models interaction outputs

tab78 <-
svyglm(malprev~1+h.number+a.newspap+a.radio+currently.w+place.deliver+sexheadhouse+a.radio:h.nu
mber+a.radio:a.newspap+currently.w:h.number

               +currently.w:a.radio+place.deliver:h.number,dw2,family = quasibinomial()

)

diabetes_results %>%

  roc_curve(truth = diabetes, .pred_pos) %>%

  autoplot()
```

```
ROC(form=malprev~1+h.number+a.newspap+a.radio+currently.w+place.deliver+sexheadhouse+a.radio:h
.number+a.radio:a.newspap+currently.w:h.number

    +currently.w:a.radio+place.deliver:h.number,

    plot="ROC",data = dhs2018)

#################Confusion matrix/ Quality of the model/2018

malprev <- predict(tab78, type = "response", newdata =dw2[["variables"]] )

head(malprev)

table(malprev > 0.5, dhs2018$malprev)

#################################################################End_Regression

##############misclassification of the model

misclas <- (6+431)/2123#####33% of misclassification


########we opt for our first model because the new one perform not much



#Start###################################Using CART on multivariable result

#A) For 2012

tab10 <- ctree(cci ~  Wealth.quintiles + V701 +

                 Antenatal.period + Antenatal.care,data = dw1[["variables"]])


nid <- nodeids(tab10)

iid <- nid[!(nid %in% nodeids(tab10, terminal = TRUE))]

(pval <- unlist(nodeapply(tab10, ids = iid,

                          FUN = function(n) info_node(n)$p.value)))


myttnc2 <- nodeprune(tab10, ids = iid[pval > 1e-5])


plot(myttnc2)
```

```
tab10 <- as.constparty(myttnc2)
################################################Using###ggtree
ggparty(myttnc2) +
  geom_edge() +
  geom_edge_label() +
  geom_node_splitvar() +
  geom_node_plot(gglist = list(geom_bar(aes(x = "", fill = cci),
                                        position = position_fill()),
                               xlab("")),
                 # draw only one label for each axis
                 shared_axis_labels = TRUE,
                 # draw line between tree and legend
                 legend_separator = TRUE
  )

################################################################End Diao script



library(foreign)
kr18<- read.spss("D:\\MOOC\\GNKR71FL.sav",to.data.frame = TRUE)
kr18 <- subset(kr18, select = c("V001", "V005", "V007", "V012", "V013", "V021",
                                "V022","V023", "V024", "V025", "V026", "V130", "V131", "V136", "V149", 
"V151",
                                "V152", "V157", "V158", "V159", "V190","V191" ,"V218", "V225", "V501", 
"V502", "V701",
                                "V714", "V626", "V313",
                                
"B4","B5","B8","MIDX","H1","H2","H3","H5","H7","H9","H10","H11","H13","H14",
                                "H31","H32A","H32B","H32C","H32E","H32I","H32J","H32K","H32L", "H32N", 
"H32S","H32Y",
```

```
"M2A","M2B","M2C","M3A","M3B","M3C", "M13", "M14"))



#Filtrer les enfants vivants
library(nlme)
library(dplyr)
#################################################Filtering on living child
krv18 <- filter(kr18,kr18$B5=="Yes")
############################################Filtering by age more than a year
krv18 <- filter(kr18,kr18$B8>=1)

##############################################checking for missing values
sapply(krv18, function(x) sum(is.na(x)))
#######################################Recoding variables before imputation
summary(krv18$V012)
table(krv18$V012)

##Recoding for living child and household number
summary(krv18$`V218`)
table(krv18$`V218`)
summary(krv18$`V136`)
table(krv18$`V136`)

str(krv18)
summary(krv18)
sapply(krv18, function(x) sum(is.na(x)))

###############################################Reducing data sets
#############recoding some variables
```

```
levels(krv18$H2)
table(krv18$H2)
levels(krv18$H2) <- c("No", "Yes","Yes", "Yes", "No")
levels(krv18$H2)
table(krv18$H2)

levels(krv18$H3)
table(krv18$H3)
levels(krv18$H3) <- c("No", "Yes","Yes", "Yes", "No")
levels(krv18$H3)

levels(krv18$H5)
table(krv18$H5)
levels(krv18$H5) <- c("No", "Yes","Yes", "Yes", "No")
levels(krv18$H5)
table(krv18$H5)


levels(krv18$H7)
table(krv18$H7)
levels(krv18$H7) <- c("No", "Yes","Yes", "Yes", "No")
levels(krv18$H7)
table(krv18$H7)

levels(krv18$H9)
table(krv18$H9)
levels(krv18$H9) <- c("No", "Yes","Yes", "Yes", "No")
levels(krv18$H9)
table(krv18$H9)
```

```
levels(krv18$H10)
table(krv18$H10)
levels(krv18$H10) <- c("No", "Yes", "No")
table(krv18$H10)

levels(krv18$H11)
table(krv18$H11)
levels(krv18$H11) <- c("No", "Yes", "Yes", "No")
table(krv18$H11)

levels(krv18$H13)
table(krv18$H13)
levels(krv18$H13) <- c("No", "Yes", "Yes", "No")
table(krv18$H13)

levels(krv18$H14)
table(krv18$H14)
levels(krv18$H14) <- c("No", "Yes", "Yes", "No")
table(krv18$H14)

levels(krv18$H31)
levels(krv18$H31) <- c("No", "Yes", "Yes", "No")
table(krv18$H31)

########################Information access
levels(krv18$V157)
levels(krv18$V157) <- c("noaccess", "access", "access", "access")
table(krv18$V157)
```

```
levels(krv18$V158)
levels(krv18$V158) <- c("noaccess", "access", "access", "access")
table(krv18$V158)

levels(krv18$V159)
levels(krv18$V159) <- c("noaccess", "access", "access", "access")
table(krv18$V159)
levels(krv18$V502)
table(krv18$V502)
levels(krv18$V502) <-  c("single","married","single")
table(krv18$V502)

levels(krv18$V149)
levels(krv18$V149) <- c("noeducation", "primary", "primary", "secondary",
                        "secondary", "higher")
table(krv18$V149)

##########################################partner education
levels(krv18$V701)
levels(krv18$V701) <- c("noeducation","primary","secondary","higher","noeducation")
table(krv18$V701)



levels(krv18$`V152`)
levels(krv18$`V152`) <- c("16","17","18","19","20","21","22","23","24","25","26","27",
                          "28","29","30","31","32","33","34","35","36","37","38","39",
                          "40","41","42","43","44","45","46","47","48","49","50","51",
                          "52","53","54","55","56","57","58","59","60","61","62","63",
                          "64","65","66","67","68","69","70","71","72","73","74","75",
                          "76","77","78","79","80","81","82","83","84","85","86","87",
```

```
                    "88","89","90","95","97","97")

krv18$`V152`<- as.numeric(levels(krv18$`V152`))[krv18$`V152`]

table(krv18$`V152`)

summary(krv18$`V152`)

table(krv18$`V152`)


##########################################anc

table(krv18$`M14`)

levels(krv18$`M14`)

levels(krv18$`M14`) <-
c("0","1","10","11","12","13","14","15","16","2","20","3","4","5","6","7","8","9","0")

table(krv18$`M14`)

krv18$`M14` <- as.numeric(levels(krv18$`M14`)[krv18$`M14`])

table(krv18$`M14`)

###############################

summary(krv18$`M14`)

#krv12$`M14` <- cut(krv12$`M14`,breaks = c(0,1,3,9),include.lowest = TRUE)

table(krv12$`M14`)

###########################################time first anc

levels(krv18$`M13`)

table(krv18$`M13`)

levels(krv18$`M13`) <- c("0","1","10","2","3","4","5","6","7","8","9","0")

table(krv18$`M13`)

krv18$`M13` <- as.numeric(levels(krv18$`M13`)[krv18$`M13`])

table(krv18$`M13`)


summary(krv18$`M13`)

#krv18$`M13` <- cut(krv18$`M13`,breaks = c(1,3,6,9),include.lowest = TRUE, labels = c("first-
trim","second-trim","third-trim"))

table(krv18$`M13`)
```

```
#ORT#####################################################For diarrhea
krv18$ORT[krv18$H11=="Yes" & krv18$H13=="No" &krv18$H14=="No"] <- "0"
krv18$ORT[krv18$H11=="Yes" & krv18$H13=="Yes" |krv18$H14=="Yes"] <-"1"

levels(krv18$ORT)
krv18$ORT <- factor(krv18$ORT)
table(krv18$ORT)
krv18$ORT <- factor((krv18$ORT),levels = c("0","1"))
krv18$ORT<-as.numeric(levels(krv18$ORT)[krv18$ORT])
table(krv18$ORT)



#####################################CPNM is care seeking for pneumonia

krv18$CPNM[krv18$H31=="Yes" &
        krv18$H32A=="No" &
        krv18$H32B=="No" &
        krv18$H32C=="No" &
        krv18$H32E=="No" &
        krv18$H32J=="No" &
        krv18$H32L=="No" &
        krv18$H32N=="No" &
        krv18$H32S=="No" ] <-
  "0"
krv18$CPNM[krv18$H31=="Yes" &
        krv18$H32A=="Yes" |
        krv18$H32B=="Yes" |
        krv18$H32C=="Yes" |
        krv18$H32E=="Yes" |
        krv18$H32J=="Yes" |
```

```
            krv18$H32L=="Yes" |
            krv18$H32N=="Yes" |
            krv18$H32S=="Yes" ] <-
  "1"
krv18$CPNM <- factor(krv18$CPNM)
sapply(krv18, function(x) sum(is.na(x)))

##############################Removing uninteristing variables for imputation
krv12d <- krv18[-c(1:3,5,7:13,22,25:26,29:30,32,34:35,42:55)]
krv12d1 <- krv18[c(1:3,6:7,9:10,12:13,21:22,26,29,34)]
###################################Vaccination  status
library(mice)
init = mice(krv12d, maxit=0)
#init = mice(krv18d, maxit=20)

meth1 = init$method
#meth1[which(meth1=="pmm")]<-"norm.boot"
#meth1[which(meth1=="logreg")]<-"logreg.boot"

meth1
predM = init$predictorMatrix
#predM[, c("V001")] <- 0
#predM[, c("V005")] <- 0
#predM[, c("V191")] <- 0
krv12d = mice(krv12d,pred=predM,seed = 15422,m = 5)
#krv18d = mice(krv18d,pred=predM,seed = 15422,m = 10)
#imp1=mice(krv18d,print=FALSE)
################################Inspect the convergence of the algorithm
plot(krv12d,layout = c(4,4))#####based on fig we need more iterations
#########################Inspect diagnostics
```

```
densityplot(krv12d)
densityplot(krv12d, ~ CPNM+M13+M14,
            layout = c(3,2))
bwplot(krv12d)
krv12d <- complete(krv12d)
##########################################################saving workspace
#save.image(file =("D:\\MOOC\\diao1.RData"))
#########################################################Loading workspace
#load("D:\\MOOC\\diao1.RData")
str(krv12d)
sapply(krv12d, function(x) sum(is.na(x)))
summary(krv12d)
###########################################################Merging dataframes
diaoc <- cbind(krv12d1,krv12d)
sapply(diaoc, function(x) sum(is.na(x)))

#########################################Following code not check!!!!!!!!!!!!!
#write.csv2(diaoc,file = "D:\\MOOC\\diaod18.csv")

krv18 <- read.csv2("D:\\MOOC\\diaod18.csv", header = T,
                   stringsAsFactors=TRUE)
library(dplyr)
###########################################Including missing variables
#krv18 <- cbind(krv18,krv18d1[c(13)])
str(krv18)
summary(krv18)
sapply(krv18, function(x) sum(is.na(x)))

#######################################religion
library(na.tools)
```

```
levels(krv18$V130)
levels(krv18$V130) <- c("noreligion","Christian","muslim","noreligion")
krv18$V130 <- na.bootstrap(krv18$V130)
table(krv18$V130)

levels(krv18$V131)
levels(krv18$V131) <- c("guerzé","kissi","malinké","other","peulh","soussou","toma")
table(krv18$V131)

##############################################Antenatal care
krv18$ANCS[krv18$M2A=="No" &krv18$M2B=="No" & krv18$M2C=="No"] <- "0"
krv18$ANCS[krv18$M2A=="Yes" | krv18$M2B=="Yes" | krv18$M2C=="Yes"] <- "1"
table(krv18$ANCS)

table(krv18$ANCS)
levels(krv18$ANCS)
krv18$ANCS <- factor(krv18$ANCS)
krv18$ANCS <- factor((krv18$ANCS),levels = c("0","1"))
krv18$ANCS<-as.numeric(levels(krv18$ANCS)[krv18$ANCS])
table(krv18$ANCS)

##########################################Delivery/
krv18$SBA[krv18$M3A=="No" & krv18$M3B=="No" & krv18$M3C=="No"] <- "0"
krv18$SBA[krv18$M3A=="Yes" | krv18$M3B=="Yes" | krv18$M3C=="Yes"] <-"1"
table(krv18$SBA)
krv18$SBA <- factor(krv18$SBA)
levels(krv18$SBA)
krv18$SBA <- factor((krv18$SBA),levels = c("0","1"))
krv18$SBA<-as.numeric(levels(krv18$SBA)[krv18$SBA])
krv18$SBA<-as.numeric(krv18$SBA)
```

```
table(krv18$SBA)


summary(krv18$V012)
krv18$V012 <- cut(krv18$V012,breaks = c(15,18,30,40,49),include.lowest = TRUE)
summary(krv18$V218)
krv18$V218 <- cut(krv18$V218,breaks = c(1,2,4,7,13),include.lowest = TRUE)
summary(krv18$V136)
krv18$V136 <- cut(krv18$V136,breaks = c(1,5,10,38),include.lowest = TRUE)
summary(krv18$V152)
krv18$V152<- cut(krv18$V152,breaks = c(16,25,40,60,97),include.lowest = TRUE)


library(forcats)
levels(krv18$V626)
krv18$V626 <- fct_recode(krv18$V626,
                NULL = "Never had sex",
                "No"= "Unmet need for spacing",
                "No" = "Unmet need for limiting",
                "Yes"= "Using for spacing",
                "Yes"= "Using for limiting",
                NULL = "Spacing failure",
                NULL = "Limiting failure",
                NULL = "No unmet need",
                NULL = "Not married and no sex in last 30 days",
                NULL = "Infecund, menopausal")
krv18$V626 <- na.replace(krv18$V626,.na = "No")


levels(krv18$V626)
table(krv18$V626)
####################################################Family
#krv18$FPS <- NA
```

```
krv18$FPS[krv18$V626=="No"] <- "0"
krv18$FPS[krv18$V626=="Yes"] <- "1"

levels(krv18$FPS)
krv18$FPS <- factor(krv18$FPS)
table(krv18$FPS)
krv18$FPS <- factor((krv18$FPS),levels = c("0","1"))
krv18$FPS<-as.numeric(levels(krv18$FPS)[krv18$FPS])
table(krv18$FPS)

#Trois doses DTP
#krv18$DPT3 <- NA
krv18$DPT3[krv18$H3=="No" |krv18$H5=="No" |krv18$H7=="No"] <-"0"
krv18$DPT3[krv18$H3=="Yes" &krv18$H5=="Yes" & krv18$H7=="Yes"] <-"1"
levels(krv18$DPT3)
krv18$DPT3 <- factor(krv18$DPT3)
table(krv18$DPT3)
krv18$DPT3 <- factor((krv18$DPT3),levels = c("0","1"))
krv18$DPT3<-as.numeric(levels(krv18$DPT3)[krv18$DPT3])
table(krv18$DPT3)

#Rougole
#krv18dddd$MSL <- NA
krv18$MSL[krv18$H9=="No"] <- "0"
krv18$MSL[krv18$H9=="Yes"] <- "1"

levels(krv18$MSL)
krv18$MSL <- factor(krv18$MSL)
table(krv18$MSL)
krv18$MSL <- factor((krv18$MSL),levels = c("0","1"))
```

```
krv18$MSL<-as.numeric(levels(krv18$MSL)[krv18$MSL])
table(krv18$MSL)

#BCG
#krv18dddd$BCG <- krv18dddd$H2
#krv18dddd$BCG <- NA
krv18$BCG[krv18$H2=="No"] <-"0"
krv18$BCG[krv18$H2=="Yes"] <-"1"
table(krv18$BCG)
levels(krv18$BCG)
krv18$BCG <- factor(krv18$BCG)
table(krv18$BCG)
krv18$BCG <- factor((krv18$BCG),levels = c("0","1"))
krv18$BCG<-as.numeric(levels(krv18$BCG)[krv18$BCG])
table(krv18$BCG)




############################################Last filtering with family planning
krv18d1<- filter(krv18,krv18$FPS==0)
krv18d2<- filter(krv18,krv18$FPS==1)

####################################################Merging the two dataframes
krv18 <- rbind(krv18d1,krv18d2)

###ICC
attach(krv18)
#detach(krv18d)
#krv18d$a <- FPS
```

```
#summary(krv18d$a)

krv18$b <- (SBA+ANCS)/2

summary(krv18$b)

krv18$c <- ((2*DPT3)+MSL+BCG)/4

summary(krv18$c)

krv18$d <- (ORT+CPNM)/2

summary(krv18$d)


krv18$icc <- 0.25*(krv18$FPS+krv18$b+krv18$c+krv18$d)

krv18$icc100 <- (krv18$icc)*100

str(krv18)

summary(krv18$icc100)

################################################Filtering for diarrhea and cough

krv18n <- filter(krv18,krv18$ORT==1&krv18$CPNM==1)

####################################################For either diarrhea or cough

krv18n1 <- filter(krv18,krv18$ORT==1|krv18$CPNM==1)

#############################################################cleaning dataframes

diao18 <- krv18n1[-c(1,12,14:15,17,32:44,38:44,55:57)]


########################################################Recoding before weighting

############################Will for pregnancy

levels(diao18$V225)

levels(diao18$V225) <- c("no","no","yes")

table(diao18$V225)


summary(diao18$M13)

diao18$M13 <- cut(diao18$M13,breaks = c(0,3,6,10),include.lowest = TRUE,labels = c("first-trim","second-trim","third-trim"))

table(diao18$M13)

summary(diao18$M14)
```

```
diao18$M14 <- cut(diao18$M14,breaks = c(0,3,20),include.lowest =
TRUE,labels=c("less.than.4ANC","at.least.4ANC"))

table(diao18$M14)


diao18[c(29:36)] <- lapply(diao18[c(29:36)],factor)


levels(diao18$ORT)

levels(diao18$ORT) <- c("no","yes")

levels(diao18$CPNM) <-c("no","yes")

levels(diao18$ANCS) <- c("no","yes")

levels(diao18$FPS)  <- c("no","yes")

levels(diao18$DPT3)<- c("no","yes")

levels(diao18$SBA)<- c("no","yes")

levels(diao18$BCG)<- c("no","yes")

levels(diao18$MSL)<- c("no","yes")

diao18$V007 <- factor(diao18$V007)

diao18$icc <- cut(diao18$icc,breaks = c(0,0.49,1),include.lowest = TRUE)

table(diao18$icc)

levels(diao18$icc) <- c("<50",">=50")

diao18$icc <- relevel(diao18$icc,ref ="<50" )

#diao18$icc <- relevel(diao18$icc,ref =">=50" )


diao18$V190 <- relevel(diao18$V190,ref = "Richest")


library(questionr)

diao18 <- rename.variable(diao18,"icc","cci")


################################################################################

diao18 <- rename.variable(diao18,"V190","Wealth.quintiles")

diao18 <- rename.variable(diao18,"M14","Antenatal.care")
```

```
diao18 <- rename.variable(diao18,"V714","currently.working")

diao18 <- rename.variable(diao18,"V158","Radio")

###################### complexe design

################Descriptive statistics with weighting

library(dplyr)

library(survey)

library(party)

library(gtsummary)

library(car)

library(ggplot2)

library(GGally)

library(forestmodel)

library(Epi)

library(ggeffects)

library(ggparty)

library(intsvy)

library(questionr)

#######################################Variable Weighting

#Wt <- krv18$V005/1000000

#krv18 <-cbind(krv18,Wt)

diao18 <- rename.variable(diao18,"Wealth.quintiles","V190")

diao18 <- rename.variable(diao18,"Radio","V158")

diao18 <- rename.variable(diao18,"Antenatal.care","M14")

Wt <- diao18$V005/1000000

krv12 <-cbind(diao18,Wt)

###################### echantillon Complexe

dw1 <-svydesign(ids=~V021,data = krv12 ,strata=~V022, weights=~Wt,nest = TRUE)
```

```
 ##################################Descriptive summary
tab2018 <- tbl_svysummary(dw1)
tab2018
tab2018 <- tbl_svysummary(dw1,by="cci")
tab2018 <- add_p(tab2018)
##################################################Merging
tbl_merge(tbls = list(tab2012, tab2018)) %>%
  modify_spanning_header(everything() ~ NA_character_)

##############Excluding some variables before univariate analysis
diao181 <- diao18[-c(1,3,6:9,20,26,29:36)]
#names(diao181) <- c("V005", "V021", "V022", "V130", "V131", "V190", "V502", "V012",
#                   "V136", "V149", "V151", "V152", "V157", "V158", "V159", "V218",
#                   "V225", "V701", "V714", "B4", "M13", "M14", "cci", "icc100")
Wt <- diao181$V005/1000000
krv12 <-cbind(diao181,Wt)

####################### echantillon Complexe
dw2 <-svydesign(ids=~V021,data = krv12 ,strata=~V022, weights=~Wt,nest = TRUE)

##b)  2012
tab2018 <- tbl_svysummary(dw2,by="cci")
tab2018

tab2 <- add_p(tab2018,
              test = list(
                all_continuous() ~ "svy.t.test",
                all_categorical() ~ "svy.wald.test"
              )
```

```
)
tab2018

########################Mergin 2012 and 2018
tbl_merge(tbls = list(tab1, tab2)) %>%
  modify_spanning_header(everything() ~ NA_character_)

####################################Only use for mapping purpose
diao18 <- diao18[-c(3,6:7,11,26,29:36)]
names(diao18) <- c("V005", "V021", "V022", "V130", "V131", "V190", "V502", "V012",
                 "V136", "V149", "V151", "V152", "V157", "V158", "V159", "V218",
                 "V225", "V701", "V714", "B4", "M13", "M14", "cci", "icc100")
diao1 <- factor(diao1$V001)

##############################################Multivariate analysis
############For 2018
dput(names(diao181))
tab6 <- svyglm(cci~B4+V190+V502+V136+V151+V149+V152+V136+V157+V158+V159+V218+
                V225+V701+currently.working+M13+M14,dw2,family = quasibinomial()
)
###################Second models after taking into account collinearity
#tab6 <- svyglm(icc~B4+V190+V502+V136+V151+V149+V152+V157+V158+V218+
#                 V225+V701+V714+M13+M14,dw1,family = quasibinomial()
#)
#######################################Step AIC for choosing best model
tab8 <- step(tab6)

###############################Graphical representation of the model
ggcoef_model(tab7, exponentiate = TRUE)
forest_model(tab7)
```

```
##################Variables effects for 2012/predicted values
ggeffect(tab7,"V149")
ggeffect(tab7,"V152")
ggeffect(tab7, "V190")


#################Confusion matrix/ Quality of the model/2012
icc <- predict(tab7, type = "response", newdata =dw1[["variables"]] )
head(cci)
table(cci > 0.5, diao18$icc)
##############misclassification of the model
misclas <- (352+535)/3005#####29% of misclassification

############################################################Model Evaluation
############## ROC curve
ROC(form=cci ~  B4 + V190 + V151 + V149 + V152 +V158+ V225 + V701 +
   M13 + M14,plot="ROC",data = dw1[["variables"]])
#ROC(form=icc ~B4 + V190 + V151 + V149 + V152 + V225 + V701 +
#♣     V714 + M13 + M14,plot="ROC",data = dw1[["variables"]])
###################################################coefficients significance
anova(tab7, test = "Chisq")
##############model assessment
with(tab7,pchisq(null.deviance-deviance,df.null-df.residual,lower.tail = F))

#############################################mulitcollinearity
vif(tab7)##########no collinearity
#############################Merging univariate and multivariate analysis
#########################################
tab7 <- tab7 %>%
  tbl_regression(exponentiate = TRUE) %>%
```

```
  add_global_p(type = "II")
tab7
####################################Using gtsummary
tab8 <- tbl_regression(tab8,exponentiate = TRUE)

########## Find why second-time women used more malprev measure than 1st
tab1 <- prop.table(table(dhs$times.1ANC,dhs$w.quitile),2)
########################################Merging Multivariate data
tbl_merge(tbls = list(tab7, tab8)) %>%
  modify_spanning_header(everything() ~ NA_character_)
##########################################################Testing interactions
#a) dhs 2012 models interactions
library(glmulti)
diao18 <- dw1[["variables"]]
tab77 <- glmulti(cci ~ B4 + V190 + V502 + V151 + V149 + V152 + V157 + V158 + V218 +
                   V225 + V714 + M13,data = diao18,family=binomial())

#a) Models interaction outputs
tab77 <-svyglm(cci~1+B4+V190+V151+V149+V152+V151:B4+V149:V151+V152:B4,dw1,family =
quasibinomial())

ROC(form=icc~1+B4+V190+V151+V149+V152+V151:B4+V149:V151+V152:B4,
    plot="ROC",data = diao182012)
ggcoef_model(tab77, exponentiate = TRUE)
forest_model(tab77)
#################Confusion matrix/ Quality of the model/2012
malprev <- predict(tab77, type = "response", newdata =dw1[["variables"]] )
head(malprev)
table(malprev > 0.5, dhs2012$malprev)
##############misclassification of the model
```

```
misclas <- (64+199)/781#####33% of misclassification

########we opt for our first model because the new one perform not much



##########################b) dhs 2018 models interactions

diao18 <- dw1[["variables"]]

tab78 <- glmulti(cci ~ V190 + V151 + V149 + V152 + V157 + V218 + V225 + V701 +

                  M13,data = dhs2018,family=binomial())


#a) Models interaction outputs

tab78 <-
svyglm(malprev~1+h.number+a.newspap+a.radio+currently.w+place.deliver+sexheadhouse+a.radio:h.nu
mber+a.radio:a.newspap+currently.w:h.number

               +currently.w:a.radio+place.deliver:h.number,dw2,family = quasibinomial()

)

diabetes_results %>%

  roc_curve(truth = diabetes, .pred_pos) %>%

  autoplot()



ROC(form=malprev~1+h.number+a.newspap+a.radio+currently.w+place.deliver+sexheadhouse+a.radio:h
.number+a.radio:a.newspap+currently.w:h.number

    +currently.w:a.radio+place.deliver:h.number,

    plot="ROC",data = dhs2018)

#################Confusion matrix/ Quality of the model/2018

malprev <- predict(tab78, type = "response", newdata =dw2[["variables"]] )

head(malprev)

table(malprev > 0.5, dhs2018$malprev)

####################################################################End_Regression

##############misclassification of the model
```

```
misclas <- (6+431)/2123#####33% of misclassification

########we opt for our first model because the new one perform not much



#Start####################################Using CART on multivariable result
#A) For 2012
tab10 <- ctree(cci~Wealth.quintiles +V151+V152+V157+V158+V159+V225+currently.working
               +M13+Antenatal.care,data = dw2[["variables"]])

nid <- nodeids(tab10)
iid <- nid[!(nid %in% nodeids(tab10, terminal = TRUE))]
(pval <- unlist(nodeapply(tab10, ids = iid,
                       FUN = function(n) info_node(n)$p.value)))

myttnc2 <- nodeprune(tab10, ids = iid[pval > 1e-5])

plot(myttnc2)
tab10 <- as.constparty(myttnc2)

#################################################Using###ggtree
ggparty(tab10) +
  geom_edge() +
  geom_edge_label() +
  geom_node_splitvar() +
  geom_node_plot(gglist = list(geom_bar(aes(x = "", fill = cci),
                                    position = position_fill()),
                               xlab("")),
               # draw only one label for each axis
               shared_axis_labels = TRUE,
```

```
            # draw line between tree and legend
            legend_separator = TRUE
 )
```

```
##########################Loading libraries
library(sf)
#library(sp)
library(dplyr)
library(ggplot2)
library(tmap)
library(rgeoda)
library(spdep)


#######################################################################For 2012
Guinea_12 <- st_read("D:\\MOOC\\shps_2012\\sdr_subnational_boundaries.shp",layer =
"sdr_subnational_boundaries")
cci12 <- read.csv2 ("D:\\MOOC\\tab12spat.csv")
cci12$cci_12 <- cci12$cci_12*100
cci12$region <- factor(cci12$region)
levels(cci12$region) <- c(
"Boké","Conakry","Faranah","Kankan","Kindia","Labé","Mamou","Nzérékoré")

################################merging
OA.Census_12 <- inner_join(Guinea_12, cci12, by = c("DHSREGFR" = "region"))

######################################################################For 2018
Guinea_18 <- st_read("D:\\MOOC\\shps_2018\\sdr_subnational_boundaries.shp",layer =
"sdr_subnational_boundaries")
```

```
cci18 <- read.csv2 ("D:\\MOOC\\tab18spat.csv")

cci18$cci_18 <- cci18$cci_18*100

cci18$region <- factor(cci18$region)

levels(cci18$region) <- c(
"Boké","Conakry","Faranah","Kankan","Kindia","Labé","Mamou","N'Zérékoré")


##############################merging

OA.Census_18 <- inner_join(Guinea_18, cci18, by = c("DHSREGFR" = "region"))


#################################################################Making map for 2018

cci2012<- tm_shape(OA.Census_12) +

  tm_fill(col = "cci_12", title = "% of partial cci in 2012") +

  tm_compass(type ="arrow",position = "right") +

  tm_scale_bar(position = "center")+

  tm_text("DHSREGFR", size =0.80,col = "black")+

  tm_layout(bg.color = "skyblue", inner.margins = c(0, .02, .02, .02))+

  tm_add_legend(type = "symbol")+tm_borders(col = "white")+

  tm_xlab(text = "source= spatialdata.dhsprogram.com")

cci2012

######################################################################For 2018

cci2018  <- tm_shape(OA.Census_18) +

  tm_fill(col = "cci_18", title = "% of Partial cci in 2018") +

  tm_compass(type ="arrow",position = "right") +

  tm_scale_bar(position = "center")+

  tm_text("DHSREGFR", size =0.80,col = "black")+

  tm_layout(bg.color = "skyblue", inner.margins = c(0, .02, .02, .02))+

  tm_add_legend(type = "symbol")+tm_borders(col = "white")+

  tm_xlab(text = "source= spatialdata.dhsprogram.com")

cci2018
```

```
tmap_arrange(cci2012,cci2018,nrow = 1)



#################################################################Cluster analysis Moran
###################Working with rgeoda

########3.1 Queen Contiguity Weights
queen_weights(sf_obj, order=1, include_lower_order = False, precision_threshold = 0)
queen_w_12 <- queen_weights(OA.Census_12)
queen_w_18 <- queen_weights(OA.Census_18)

summary(queen_w_12)
summary(queen_w_18)

###################Attributes of Weight object
is_symmetric(queen_w_12)
has_isolates(queen_w_18)
weights_sparsity(queen_w_12)
weights_sparsity(queen_w_18)

###To access the details of the weights:list the neighbors of a specified observation
nbrs <- get_neighbors(queen_w_12, idx = 1)
cat("\nNeighbors of the 1-st observation are:", nbrs)

#To compute the spatial lag of a specified observation by passing the values of the selected variable
lag12 <- spatial_lag(queen_w_12, OA.Census_12['cci_12'])
lag12
lag18 <- spatial_lag(queen_w_18, OA.Census_18['cci_18'])
lag18
#############3.2 Rook Contiguity Weights
```

```
### to create a Rook contiguity weights using the sf object guerry
rook_w <- rook_weights(OA.Census_12)
summary(rook_w)

#######The weights we created are in memory. To save the weights to a file, one can call the function

#save_weights(rook_w, OA.Census['ID_1'], out_path = 'D:\\MOOC\\OA.Census_r.gal',
#            layer_name = 'NAME_1')

############3.3 Distance Based Weights
#min_distthreshold(GeoDa gda, bool is_arc = False, is_mile = True)
##To create a Distance based weights, one can call the function `distance_weights`

###Then, with this distance threshold, we can create a distance-band weights using the function
#distance_weights(geoda_obj, dist_thres, power=1.0,  is_inverse=False, is_arc=False, is_mile=True)

dist_thres <- min_distthreshold(OA.Census_12)
dist_thres

#dist_w <- distance_weights(OA.Census_12, dist_thres)
#summary(dist_w)

############3.4 K-Nearest Neighbor Weights
#knn_weights(gda, k, power = 1.0,is_inverse = False, is_arc = False, is_mile = True)

##to create a 6-nearest neighbor weights using Guerry
knn6_w <- knn_weights(OA.Census_12, 6)
summary(knn6_w)
```

```
##To create a kernel weights with fixed bandwith
bandwidth <- min_distthreshold(OA.Census_12)
#kernel_w <- kernel_weights(OA.Census_12, bandwidth, kernel_method = "uniform")
#summary(kernel_w)

########To create a kernel weights with adaptive bandwidth or using max Knn distance as bandwidth

#adptkernel_w = kernel_knn_weights(OA.Census_12, 6, "uniform")

#summary(adptkernel_w)

###Local Indicators of Spatial Association–LISA
#guerry_path <- system.file("extdata", "Guerry.shp", package = "rgeoda")
#guerry <- st_read(guerry_path)
cci12 <- OA.Census_12["cci_12"]
cci18 <- OA.Census_18["cci_18"]

lisa12 <- local_moran(queen_w_12, cci12)
lisa18 <- local_moran(queen_w_18, cci12)

#######we can call the function lisa_values() to get the values of the local Moran
lms12 <- lisa_values(gda_lisa = lisa12)
lms12
lms18 <- lisa_values(gda_lisa = lisa18)
lms18
##To get the pseudo-p values of significance of local Moran computation

pvals12 <- lisa_pvalues(lisa12)
pvals12
```

```
###To get the cluster indicators of local Moran computation
##which can be accessed via the function lisa_labels()

lbls12 <- lisa_labels(lisa12)
lbls12

###re-run the above local Moran example using 9,999 permutations.
set.seed(123)
lisa12 <- local_moran(queen_w_12, cci12, permutations = 9999)
lisa18 <- local_moran(queen_w_18, cci18, permutations = 9999)
###############Get pvalues
pvals12 <- lisa_pvalues(lisa12)
pvals12
pvals18 <- lisa_pvalues(lisa18)
pvals18

#rgeoda uses GeoDa C++ code, in which multi-threading is used to accelerate the computation of LISA. We can use the argument
#ncpu to specify how many threads to run the computation

lisa2012 <- local_moran(queen_w_12, cci12, cpu_threads = 4)
lisa2018 <- local_moran(queen_w_18, cci18, cpu_threads = 4)

###Get the False Discovery Rate value based on current pseudo-p values
fdr12 <- lisa_fdr(lisa2012, 0.05)
fdr12
fdr18 <- lisa_fdr(lisa2018, 0.05)
fdr18
#Then, one can set the FDR value as the cutoff p-value to filter the cluster results:
cat_fdr12 <- lisa_clusters(lisa2012, cutoff = fdr12)
```

```
cat_fdr12
cat_fdr18 <- lisa_clusters(lisa2018, cutoff = fdr18)
cat_fdr18

############Local geary
##For example, we can call the function local_geary() with the created
#Queen weights and the data "crm_prp" as input parameters
cci_2012 <- local_geary(queen_w_12, cci12)
cci_2018 <- local_geary(queen_w_18, cci18)

##To get the cluster indicators of the local Geary computation:
lisa_clusters(cci_2012)
lisa_clusters(cci_2018)

##To get the pseudo-p values of the local Geary computation
lisa_pvalues(cci_2012)
lisa_pvalues(cci_2018)

###6.3 Create Local Moran Map
lisa_colors <- lisa_colors(lisa12)
lisa_labels <- lisa_labels(lisa12)
lisa_clusters <- lisa_clusters(lisa12)

loclis12<- plot(st_geometry(OA.Census_12),
        col=sapply(lisa_clusters, function(x){return(lisa_colors[[x+1]])}),
        border = "#333333", lwd=0.2)
title(main = "Local Moran Map of cci  2012")
legend('bottomleft', legend = lisa_labels, fill = lisa_colors, border = "#eeeeee")

############For 2018
```

```
lisa_colors <- lisa_colors(lisa18)
lisa_labels <- lisa_labels(lisa18)
lisa_clusters <- lisa_clusters(lisa18)

loclis18<-plot(st_geometry(OA.Census_18),
               col=sapply(lisa_clusters, function(x){return(lisa_colors[[x+1]])}),
               border = "#333333", lwd=0.2)
title(main = "Local Moran Map of cci 2018")
legend('bottomleft', legend = lisa_labels, fill = lisa_colors, border = "#eeeeee")


################################
lisa_p <- lisa_pvalues(lisa12)
p_labels <- c("Not significant", "p <= 0.05", "p <= 0.01", "p <= 0.001")
p_colors <- c("#eeeeee", "#84f576", "#53c53c", "#348124")
plot(st_geometry(OA.Census_12),
     col=sapply(lisa_p, function(x){
       if (x <= 0.001) return(p_colors[4])
       else if (x <= 0.01) return(p_colors[3])
       else if (x <= 0.05) return (p_colors[2])
       else return(p_colors[1])
     }),
     border = "#333333", lwd=0.2)
title(main = "Local Moran Map of cci 2012")
legend('bottomleft', legend = p_labels, fill = p_colors, border = "#eeeeee")


#####################################
lisa_p <- lisa_pvalues(lisa18)
p_labels <- c("Not significant", "p <= 0.05", "p <= 0.01", "p <= 0.001")
```

```
p_colors <- c("#eeeeee", "#84f576", "#53c53c", "#348124")
plot(st_geometry(OA.Census_18),
     col=sapply(lisa_p, function(x){
       if (x <= 0.001) return(p_colors[4])
       else if (x <= 0.01) return(p_colors[3])
       else if (x <= 0.05) return (p_colors[2])
       else return(p_colors[1])
     }),
     border = "#333333", lwd=0.2)
title(main = "Local Moran Map of cci 2018")
legend('bottomleft', legend = p_labels, fill = p_colors, border = "#eeeeee")


#####################################################working with spded
#)A For 2012 survey Global Moran
#Extracting neighbors(according to Queen)
#9.1 Find queen neighbors
neighbours <- poly2nb(OA.Census_12)
neighbours
neighbours1 <- poly2nb(OA.Census_18)
neighbours1
#Crating weight matrix
neighbours11 <- nb2listw(neighbours,zero.policy=TRUE)
moran.test(OA.Census_12$cci_12, neighbours11)

neighbours2 <- nb2listw(neighbours1,zero.policy=TRUE)
moran.test(OA.Census_18$cci_18, neighbours2)

#neighbours11 |> nb2listw(style="B") -> lw_q_B
#neighbours2 |> nb2listw(style="W") -> lw_q_W
```

```
########################
glance_htest <- function(ht) c(ht$estimate,
                              "Std deviate"=unname(ht$statistic),
                              "p.value"=unname(ht$p.value))

(OA.Census_12 |>
    st_drop_geometry() |>
    subset(select=DHSREGFR, drop=TRUE) -> Types) |>
  table()



(OA.Census_12 |>
    st_drop_geometry() |>
    subset(select=cci_12, drop=TRUE) -> z) |>
  moran.test(listw=neighbours11, randomisation=FALSE) |>
  glance_htest()

(OA.Census_18 |>
    st_drop_geometry() |>
    subset(select=cci_18, drop=TRUE) -> z1) |>
  moran.test(listw=neighbours2, randomisation=FALSE) |>
  glance_htest()

###########Boostrap compute
(z |>
    moran.test(listw=neighbours11) -> mtr) |>
  glance_htest()

(z1 |>
    moran.test(listw=neighbours2) -> mtr1) |>
```

```
  glance_htest()


set.seed(1)
z |>
  moran.mc(listw=neighbours11, nsim=999, return_boot = TRUE) -> mmc


z1 |>
  moran.mc(listw=neighbours2, nsim=999, return_boot = TRUE) -> mmc1


c("Permutation bootstrap"=var(mmc$t),
  "Analytical randomisation"=unname(mtr$estimate[3]))


c("Permutation bootstrap"=var(mmc1$t),
  "Analytical randomisation"=unname(mtr1$estimate[3]))


#####################
# MORAN scatter plot
z |> moran.plot(listw=neighbours11, labels=OA.Census_12$ID_2, cex=1, pch=".", xlab="malprev12", ylab="lagged malprev12") -> infl_W1
z1 |> moran.plot(listw=neighbours2, labels=OA.Census_18$ID_2, cex=1, pch=".", xlab="malprev18", ylab="lagged malprev18") -> infl_W2


#####################Produce map
OA.Census_12$hat_value <- infl_W1$hat
tm_shape(OA.Census_12) + tm_fill("hat_value")


OA.Census_18$hat_value <- infl_W2$hat
tm_shape(OA.Census_18) + tm_fill("hat_value")
##############################
z |>
  localmoran(listw=neighbours11, conditional=FALSE, alternative="two.sided") -> locm
```

```
z1 |>
  localmoran(listw=neighbours2, conditional=FALSE, alternative="two.sided") -> locm1

####################
pva <- \(pv) cbind("none"=pv, "bonferroni"=p.adjust(pv, "bonferroni"), "fdr"=p.adjust(pv, "fdr"), "BY"=p.adjust(pv, "BY"))
locm |>
  subset(select="Pr(z != E(Ii))", drop=TRUE) |>
  pva() -> pvsp
f <- \(x) sum(x < 0.05)
apply(pvsp, 2, f)

pva <- \(pv) cbind("none"=pv, "bonferroni"=p.adjust(pv, "bonferroni"), "fdr"=p.adjust(pv, "fdr"), "BY"=p.adjust(pv, "BY"))
locm1 |>
  subset(select="Pr(z != E(Ii))", drop=TRUE) |>
  pva() -> pvsp
f <- \(x) sum(x < 0.05)
apply(pvsp, 2, f)
#####################
library(parallel)
set.coresOption(ifelse(detectCores() == 1, 1, detectCores()-1L))
###################
system.time(z |>
        localmoran_perm(listw=neighbours11, nsim=499, alternative="two.sided", iseed=1) -> locm_p)

system.time(z1 |>
        localmoran_perm(listw=neighbours2, nsim=499, alternative="two.sided", iseed=1) -> locm_p1)

#################
brks <- qnorm(c(0, 0.00001, 0.0001, 0.001, 0.01, 0.025, 0.5, 0.975, 0.99, 0.999, 0.9999, 0.99999, 1))
```

```
(locm_p |>
    subset(select=Z.Ii, drop=TRUE) |>
    cut(brks) |>
    table()-> tab)
###############
z |>
  localmoran(listw=neighbours11, conditional=TRUE, alternative="two.sided") -> locm_c

z1 |>
  localmoran(listw=neighbours2, conditional=TRUE, alternative="two.sided") -> locm_c1

###################
OA.Census_12$locm_Z <- locm[, "Z.Ii"]
OA.Census_12$locm_c_Z <- locm_c[, "Z.Ii"]
OA.Census_12$locm_p_Z <- locm_p[, "Z.Ii"]
#liss12<- tm_shape(OA.Census_12) + tm_fill(c("locm_Z", "locm_c_Z", "locm_p_Z"), breaks=brks, midpoint=0, title="Standard deviates of\nLocal Moran's I") + tm_facets(free.scales=FALSE, ncol=2) + tm_layout(panel.labels=c("Analytical total", "Analytical conditional", "Conditional permutation"))
tm_shape(OA.Census_12) + tm_fill(c("locm_Z", "locm_c_Z", "locm_p_Z"), breaks=brks, midpoint=0, title="Standard deviates of\nLocal Moran's I") + tm_facets(free.scales=FALSE, ncol=2) + tm_layout(panel.labels=c("Analytical total", "Analytical conditional", "Conditional permutation"))

OA.Census_18$locm_Z1 <- locm1[, "Z.Ii"]
OA.Census_18$locm_c_Z1 <- locm_c1[, "Z.Ii"]
OA.Census_18$locm_p_Z1 <- locm_p1[, "Z.Ii"]
#liss18<- tm_shape(OA.Census_18) + tm_fill(c("locm_Z1", "locm_c_Z1", "locm_p_Z1"), breaks=brks, midpoint=0, title="Standard deviates of\nLocal Moran's I") + tm_facets(free.scales=FALSE, ncol=2) + tm_layout(panel.labels=c("Analytical total", "Analytical conditional", "Conditional permutation"))
tm_shape(OA.Census_18) + tm_fill(c("locm_Z1", "locm_c_Z1", "locm_p_Z1"), breaks=brks, midpoint=0, title="Standard deviates of\nLocal Moran's I") + tm_facets(free.scales=FALSE, ncol=2) + tm_layout(panel.labels=c("Analytical total", "Analytical conditional", "Conditional permutation"))
#tmap_arrange(liss12,liss18,nrow = 2)
#####################
```

```
q_mean <- attr(locm, "quadr")$mean

OA.Census_12$hs_ac_q <- OA.Census_12$hs_cp_q <- OA.Census_12$hs_an_q <- q_mean

is.na(OA.Census_12$hs_an_q) <- !(OA.Census_12$locm_Z < brks[6] | OA.Census_12$locm_Z > brks[8])

is.na(OA.Census_12$hs_cp_q) <- !(OA.Census_12$locm_p_Z < brks[2] | OA.Census_12$locm_p_Z > brks[12])

is.na(OA.Census_12$hs_ac_q) <- !(OA.Census_12$locm_c_Z < brks[2] | OA.Census_12$locm_c_Z > brks[12])


#hotp12<- tm_shape(OA.Census_12) + tm_fill(c("hs_an_q", "hs_ac_q", "hs_cp_q"), colorNA="grey95", textNA="Not significant", title="Incomplete MPM hotspot status\nLocal Moran's I") + tm_facets(free.scales=FALSE, ncol=2) + tm_layout(panel.labels=c("Unadjusted analytical total", "Bonferroni analytical cond.", "Cond. perm. with Bonferroni"))

tm_shape(OA.Census_12) + tm_fill(c("hs_an_q", "hs_ac_q", "hs_cp_q"), colorNA="grey95", textNA="Not significant", title="Incomplete MPM hotspot status\nLocal Moran's I") + tm_facets(free.scales=FALSE, ncol=2) + tm_layout(panel.labels=c("Unadjusted analytical total", "Bonferroni analytical cond.", "Cond. perm. with Bonferroni"))

###############

q_mean <- attr(locm1, "quadr")$mean

OA.Census_18$hs_ac_q <- OA.Census_18$hs_cp_q <- OA.Census_18$hs_an_q <- q_mean

is.na(OA.Census_18$hs_an_q) <- !(OA.Census_18$locm_Z1 < brks[6] | OA.Census_18$locm_Z > brks[8])

is.na(OA.Census_18$hs_cp_q) <- !(OA.Census_18$locm_p_Z1 < brks[2] | OA.Census_18$locm_p_Z > brks[12])

is.na(OA.Census_18$hs_ac_q) <- !(OA.Census_18$locm_c_Z1 < brks[2] | OA.Census_18$locm_c_Z > brks[12])


#hotp18<- tm_shape(OA.Census_18) + tm_fill(c("hs_an_q", "hs_ac_q", "hs_cp_q"), colorNA="grey95", textNA="Not significant", title="Incomplete MPM hotspot status\nLocal Moran's I") + tm_facets(free.scales=FALSE, ncol=2) + tm_layout(panel.labels=c("Unadjusted analytical total", "Bonferroni analytical cond.", "Cond. perm. with Bonferroni"))

tm_shape(OA.Census_18) + tm_fill(c("hs_an_q", "hs_ac_q", "hs_cp_q"), colorNA="grey95", textNA="Not significant", title="Incomplete MPM hotspot status\nLocal Moran's I") + tm_facets(free.scales=FALSE, ncol=2) + tm_layout(panel.labels=c("Unadjusted analytical total", "Bonferroni analytical cond.", "Cond. perm. with Bonferroni"))

tmap_arrange(hotp12,hotp18,nrow = 2)
```

```
#save.image(file =("D:\\MOOC\\diospat_v1.RData"))

load("D:\\MOOC\\diospat_v1.RData")

############################################################Interpolation_Krigging
```
